# Impact of a *G2-EPSPS* & *GAT* Dual Transgenic Glyphosate-Resistant Soybean Line on the Soil Microbial Community under Field Conditions Affected by Glyphosate Application

**DOI:** 10.1264/jsme2.ME20056

**Published:** 2020-11-07

**Authors:** Minkai Yang, Zhongling Wen, Aliya Fazal, Xiaomei Hua, Xinhong Xu, Tongming Yin, Jinliang Qi, Rongwu Yang, Guihua Lu, Zhi Hong, Yonghua Yang

**Affiliations:** 1 Institute for Plant Molecular Biology, State Key Laboratory of Pharmaceutical Biotechnology, School of Life Sciences, Nanjing University, Nanjing 210023, China; 2 Research Center for Soil Pollution Prevention and Control, Nanjing Institute of Environmental Sciences, MEE, Nanjing 210042, China; 3 Co-Innovation Center for Sustainable Forestry in Southern China, Nanjing Forestry University, Nanjing 210037, China; 4 School of Life Sciences, Huaiyin Normal University, Huaian 223300, China

**Keywords:** *G2-EPSPS* & *GAT* genes, transgenic soybean, glyphosate, root-associated bacterial community, *Ensifer fredii*

## Abstract

In the past thirty years, the biosafety of the aboveground part of crops, including horizontal gene transferal through pollen dispersal and hybridization, has been the focus of research; however, microbial communities in the underground part are attracting increasing attention. In the present study, the soybean root-associated bacterial communities of the *G2-EPSPS* plus *GAT* transgenic soybean line Z106, its recipient variety ZH10, and Z106 treated with glyphosate (Z106J) were compared at the seedling, flowering, and seed filling stages by high-throughput sequencing of the V4 hypervariable regions of 16S rRNA gene amplicons using Illumina MiSeq. The results obtained showed no significant differences in the alpha/beta diversities of root-associated bacterial communities at the three stages among ZH10, Z106, and Z106J under field growth conditions; however, the relative abundance of four main nitrogen-fixing bacterial genera significantly differed among ZH10, Z106, and Z106J. Ternary plot results indicated that in the root compartment, the proportional contributions of rhizobial nitrogen-fixing *Ensifer fredii* and *Bradyrhizobium elkanii*, which exhibit an extremely broad nodulation host range, markedly differed among the three treatments at the three stages. Thus, the present results indicate that transgenic *G2-EPSPS* and *GAT* soybean may induce different changes in functional bacterial species in soil, such as *E. fredii* and *B. elkanii*, from ZH10, which were compensated for/enriched at the flowering and seed filling stages, respectively, to some extent through as of yet unknown mechanisms by transgenic soybean treated with glyphosate.

The world’s first genetically modified (GM) plant was introduced in 1983, and the United States of America took the lead in promoting the industrialization of GM crops in 1996 (Brookes and Barfoot, 2013). After entering the 21st century, the development momentum of this industrialization has become increasingly fierce worldwide. Developed and developing countries are both increasingly focusing on the application of GM technology in agriculture, including China. After 20 years of development, China has achieved advances in the field of biotechnology research and development (Zhang *et al.*, 2018; Feng and Yang, 2019). While GM crops bring significant benefits to humans, biosafety issues, including environmental and biological factors, need to be considered (Benbrook, 2016; ISAAA, 2017; 2018). The impact of GM herbicide-resistant crops on the soil microbial ecosystem has also become the focus of GM safety assessments (Dale *et al.*, 2002; Babujia *et al.*, 2016), particularly the importance of and issues associated with the development of GM soybean in China, which is partly due to its origin.

Two approaches are currently used to obtain glyphosate-resistant soybean: the introduction of the glyphosate-tolerant 5-enolpyruvylshikimate-3-phosphate synthase (*EPSPS*) gene into GM soybeans to improving their tolerance as well as the introduction of a glyphosate-degradable gene, which degrades glyphosate before it exerts its effects, thereby reducing damage to soybean (Duke and Powles, 2008; Guo *et al.*, 2015). Many genes have been found to degrade glyphosate, and the glyphosate N-acetyltransferase (*GAT*) gene exhibits good resistance to glyphosate. Under the effects of *GAT*, glyphosate may be converted into N-acetylglyphosate, which is non-toxic to plants (Castle *et al.*, 2004).

Three soybean treatments were examined in the present study: the soybean cultivar Zhonghuang10 (ZH10) as the recipient, its *G2-EPSPS & GAT* dual transgenic soybean cultivar ZH10-6 (Z106) (Guo *et al.*, 2016), and ZH10-6 treated with glyphosate (Z106J), with the following aims: 1) to compare and analyze the composition and abundance of root-associated bacterial communities among three soybean treatments with three different sampling compartments at the seedling, flowering and seed filling stages, and 2) to elucidate the impact on the enrichment/derichment of specific bacterial species under field growth conditions using the high-throughput sequencing of 16S rRNA gene (V4 region) amplicons via the Illumina MiSeq sequencing platform.

## Materials and Methods

### Plant materials and sampling methods

The recipient soybean cultivar ZH10, the *G2-EPSPS* and *GAT* transgenic herbicide-tolerant soybean line Z106, and Z106 treated with glyphosate (Z106J) were used as three treatments in this experiment (Guo *et al.*, 2016). All soybean lines were treated with diammonium hydrogen phosphate ([NH_4_]_2_HPO_4_) 300 kg hectare^–1^, and potassium chloride (KCl) 150 kg hectare^–1^ before planting, while no fertilizer was applied during the experiment. Regarding Z106J, glyphosate was applied at 900‍ ‍g hectare^–1^ at the seedling stage. The experimental field (the Shunyi Experimental Field Station of the Crop Research Institute, CAAS) is located in Shunyi district, Beijing, China (N 40.237°, E 116.570°). This field was divided into 36 plots (5×4‍ ‍m per plot) with a randomized complete block design for national joint experiments in May 2015, and the layout and design of the experimental field is shown in Fig. S1 (Lu *et al.*, 2017), including the above three treatments with three replications. The climate conditions of the experimental location were a sub-humid warm temperate continental monsoon climate. Experimental samples were collected at the seedling stage on July 5, flowering stage on July 29, and seed filling stage on August 24 in 2015.

At all three stages, each soybean treatment was sampled from three different plots with two sampling points per plot, which were randomly distributed across the field, and composite samples from two sampling points per plot were made and treated as one replicate. Regarding the naming of samples, A represents bulk soil, *i.e.*, soil before planting, while B, C, and D represent the seedling (V4–V5), flowering (R1–R2), and seed filling (R5–R6) stages, respectively. The SO, RH, and RT compartments represent surrounding soil, rhizospheric soil, and root samples, respectively. Soil loosely adhering to the roots was shaken off the soybean plant as the surrounding soil sample (SO). The rhizospheric soil sample (RH) was collected by brushing off soil that tightly adhered to the root surface. The root sample (RT) (a mixed sample of the rhizoplane/endosphere and the layer of endophytes in roots) was collected after washing with phosphate-buffered saline (PBS) (Lu *et al.*, 2017). All samples were then stored at 4°C for a basic physicochemical analysis and at –80°C for DNA extraction (Lu *et al.*, 2018a, 2018b).

### Basic physicochemical properties of soils and plants

To analyze the carbon and nitrogen contents of surrounding soil and dried plant tissues, all samples were sent to the Modern Analysis Center of Nanjing University. The pH and water content of samples were also tested for surrounding soil. In the acetylene reduction assay (ARA) on surrounding soil, 10‍ ‍g of each sample was mixed with 1.4‍ ‍mL of 0.1 mol L^–1^ glucose in a 65‍ ‍mL conical flask, followed by an injection of 7‍ ‍mL of acetylene instead of air with a syringe and an incubation at 28°C for 15 hours before measurements of ethylene production by gas chromatography.

### DNA extraction from soil and root samples

In the present study, every biological replicate was a mixture of total metagenomic DNA extracted using approximately 0.6‍ ‍g soil from each sampling point by the Power Soil DNA Isolation Kit (MoBio Laboratories), following the instructions described by Lu *et al.* (2017) with minor modifications. In root sample preparation, 2×0.7‍ ‍g of root segments from the root tip of every biological replicate was carefully homogenized with liquid nitrogen using a mortar and pestle and then subjected to total metagenomic DNA extraction using the same method described above.

### 16S rRNA gene amplicon sequencing via the Illumina MiSeq platform

We used an improved dual-index high-throughput sequencing approach with paired-end 250 nt. A ‘heterogeneity spacer’ (0 to 7‍ ‍bp) was introduced into the index sequence, which allowed an equal proportion of samples to be sequenced (Fadrosh *et al.*, 2014). In brief, fusion primers included appropriate P5 or P7 Illumina adapter sequences, an 8-nt index sequence, and gene-specific primers for amplifying the V4 region of 16S rRNA gene (Peiffer *et al.*, 2013), namely, 515F (5′-GTGCCAGCMGCCGCGGTAA-3′) and 806R (5′-GGACTACHVGGGTWTCTAAT-3′) (Caporaso *et al.*, 2010). PCR amplification, product purification, and library quality determinations and quantification were performed as previously described by Lu *et al.* (2017). High-throughput sequencing of the qualified libraries was performed on the Illumina MiSeq platform (Illumina) with the MiSeq Reagent Kit by BGI Tech Solutions. The sequencing clean data of 90 samples have been submitted to the Sequence Read Archive (SRA) with the SRA accession number PRJNA613555.

### Analysis of 16S rRNA gene amplicon sequencing data

A total of 10,532,365 qualified paired-end clean reads with an average count per sample of 102,256 (range: 44,754–161,581) were obtained from all samples at the seedling, flowering, and seed filling stages. Total qualified paired clean reads at the seedling stage were 2,933,264 and the average count per sample was 97,775‍ ‍(range: 44,754–140,183). At the flowering stage, total qualified paired-end clean reads were 3,361,258 and the average count per sample was 112,042 (range: 50,930–156,604). At the seed filling stage, total qualified paired-end clean reads were 2,989,410 and the average count per sample was 87,924 (range: 49,695–145,012) (Table S1). Operational taxonomic units (OTUs) were selected as previously described (Lu *et al.*, 2018a, 2018b) with minor modifications. Chimeras were filtered out using UCHIME (v4.2.40) and connected tags were filtered to eliminate low quality and short sequences using QIIME (v1.7.0) before clean tags were obtained (Edgar *et al.*, 2011; Edgar, 2013). As described previously by Lu *et al.* (Lu *et al.*, 2018a, 2018b), OTU counts in each sample’s library were normalized after species annotation and phylogenetic tree construction, mitochondrion/chloroplast-related sequences (phylum *Cyanobacteria*, family *Mitochondria*) from the sequence data set were simultaneously removed, and all of the operations described above were conducted in the I-Sanger platform (http://www.i-sanger.com). Alpha and beta diversity analyses were then conducted based on OTUs and species annotation results (Wen *et al.*, 2019). In addition, the OTU rank abundance curve was drawn to reflect species richness and evenness (Fig. S2), and the Pan/Core OTU analysis was used to observe increases in total species and decreases in common species with higher sample numbers (Fig. S3).

### Alpha diversity, beta diversity, and the taxonomic analysis

**Fig. 1.**
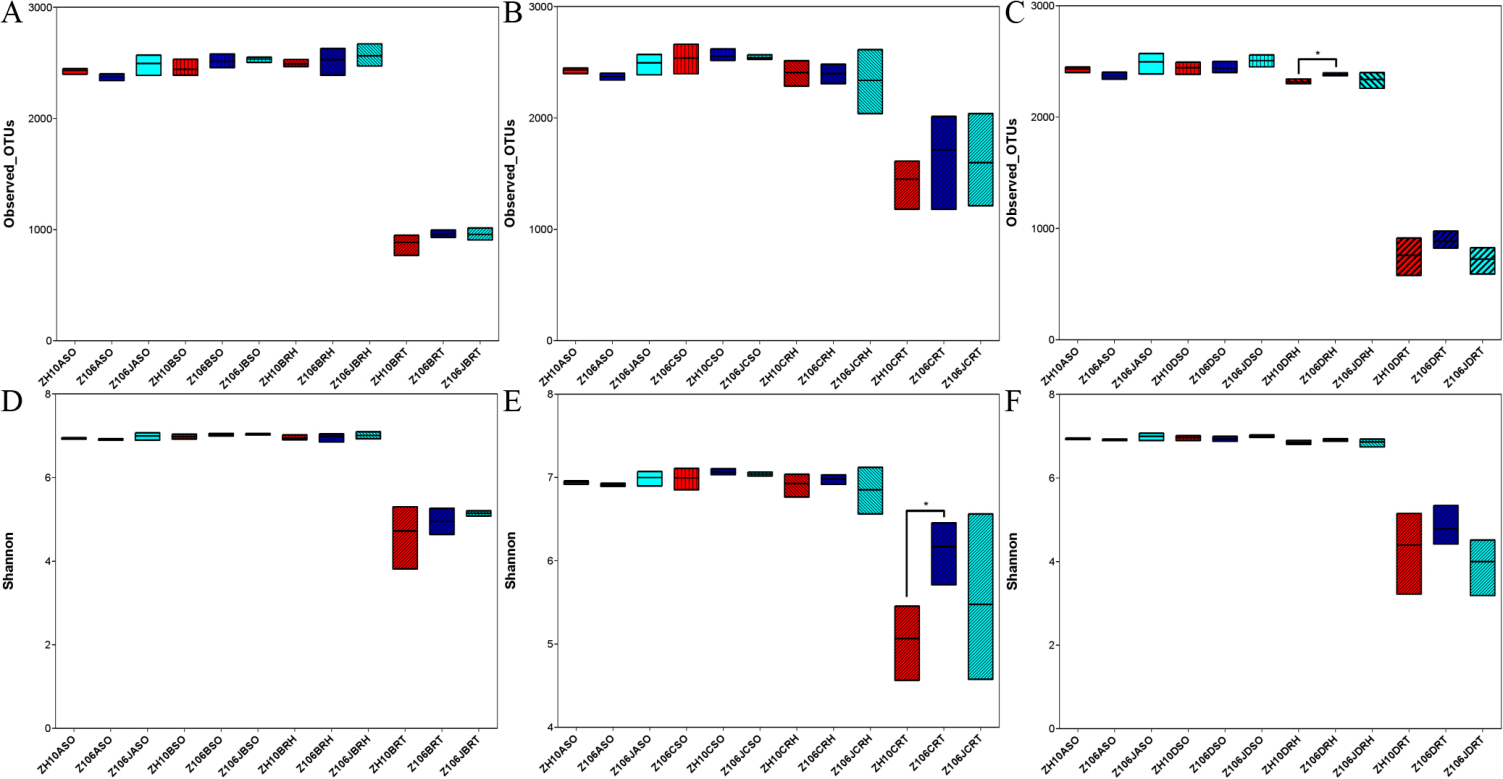
A boxplot of alpha diversity among three treatments. A) Observed OTUs index of the seedling stage; B) Observed OTUs index of the flowering stage; C) Observed OTUs index of the seed filling stage; D) Shannon index of the seedling stage; E) Shannon index of the flowering stage; F) Shannon index of the seed filling stage. Z106 and ZH10 represent the transgenic soybean (ZH10-6) and its recipient cultivar (Zhonghuang 10), respectively. Z106J represents Z106 treated with glyphosate. A represents soil before planting, B, C, and D represent the seedling, flowering, and seed filling stages, respectively. SO, RH, and RT represent surrounding soil, rhizospheric soil, and root samples, respectively. The asterisk (*, *P*<0.05) indicates a significant difference according to the Student’s *t*-test.

Alpha diversity is applied to analyses of the complexity of species diversity for a sample, which may be expressed by different indices, including observed species, Chao1, ACE, Shannon, and Simpson (Fig. 1 and S4) (Schloss *et al.*, 2009), and a boxplot of alpha diversity was made by Graphpad Prism. The beta diversity analysis is used to assess differences in species complexity in samples through several values, such as Bray-Curtis, weighted UniFrac (WUF), and unweighted UniFrac (UUF). Beta diversity was calculated by QIIME (Caporaso *et al.*, 2010) to evaluate differences in species complexity in samples among ZH10, Z106, and Z106J. A principal coordinate analysis (PCoA) based on WUF was also performed with QIIME (Peiffer *et al.*, 2013; Tian *et al.*, 2019). A Partial Least Squares Discriminant Analysis (PLS-DA) was performed with the mix Omics package of software R (Fig. S5) (Rohart *et al.*, 2017). Taxa clustering was conducted based on the relative abundance of each taxon; longitudinal clustering indicates the similarity of all taxa among different samples, while horizontal clustering indicates the similarity of certain taxa among different samples (Fig. S6) (Jie *et al.*, 2019). All data were processed in I-Sanger (Wen *et al.*, 2019). In addition, according to previous studies (Lu *et al.*, 2017; Wen *et al.*, 2019), the relative abundance of the main nitrogen-fixing bacterial genera was compared using a one-way ANOVA, and four genera that showed significant differences among the three treatments, namely, *Bradyrhizobium* (Kaneko *et al.*, 2002; de Souza *et al.*, 2012), *Bacillus* (Seldin and Dubnau, 1985; Talebi *et al.*, 2013), *Cupriavidus* (Andam *et al.*, 2007; Liu *et al.*, 2012), and *Ochrobactrum* (Ngom *et al.*, 2004; Wackerow-Kouzova, 2007), were examined in more detail.

### Ternary plot analysis

A ternary plot is an equilateral triangle describing the ratio relationship of the different attributes of three variables. In the analysis, the species composition of three groups of samples may be compared and analyzed according to species classification information. The proportion and relationship of different species in samples may be visually displayed by a triangle diagram. A ternary plot was drawn by GGTERN (http://www.ggtern.com/) (Bulgarelli *et al.*, 2012; Schlaeppi *et al.*, 2014).

### Statistical analysis

Our samples belonged to different groups, and the sample number per group was 3. The Student’s *t*-test for alpha diversity was performed in I-Sanger. An analysis of similarities (ANOSIM) and permutational MANOVA (PERMANOVA/Adonis) were complementary, non-parametric analyses that were performed using the database Silva128/16S, with/without subsampling, as well as the vegan package of software R (v3.1.3) in I-Sanger based on the Bray–Curtis, WUF, and UUF distance metrics (Schloss *et al.*, 2009; Wen *et al.*, 2019).

### Ethics statement

The Ministry of Agriculture of the People’s Republic of China issued permissions for the locations. Field studies did not involve endangered species. The experimental field was not protected or privately owned in any way.

## Results

### Analysis of basic physicochemical properties of soils and plants

According to the information obtained from the China Soil Database (http://vdb3.soil.csdb.cn/), the soil type in Shunyi district, Beijing city, is Aquic cinnamon soil. As shown in Table 1, the results of soil pH and water content showed significant differences among the three soybean treatments from the seedling to seed filling stages. The soil nitrogen content did not significantly differ among the three treatments at the three stages, while a significant difference was observed in the soil carbon content at the seed filling stage only. The soil nitrogenase activities of Z106 and Z106J were significantly higher than that of ZH10 at the seedling stage only. Significant differences were observed in the plant carbon content at the seedling and flowering stages, while the plant nitrogen content significantly differed at all three stages among the three treatments.

### Alpha diversity of root-associated bacterial communities among ZH10, Z106, and Z106J

The mean and standard deviation (SD) were calculated based on six alpha diversity indices of all replicates of the surrounding soil, rhizospheric soil, and root samples of ZH10, Z106 and Z106J, and the Student’s *t*-test was used for multigroup comparisons. The results obtained revealed no significant differences in alpha diversity among the three treatments at the three stages, except for some comparison groups in one alpha diversity index (Table S2), such as the Observed OTUs in the rhizospheric soil samples of ZH10 and Z106 at the seed filling stage, and the Shannon index of the root samples of ZH10 and Z106 at the flowering stage (Fig. 1 and S4). In addition, the Observed OTUs and Shannon index of root samples at the seedling, flowering, and seed filling stages were markedly lower than those of the surrounding soil and rhizospheric soil samples (Fig. 1).

### Beta diversity of root-associated bacterial communities among ZH10, Z106, and Z106J

**Fig. 2.**
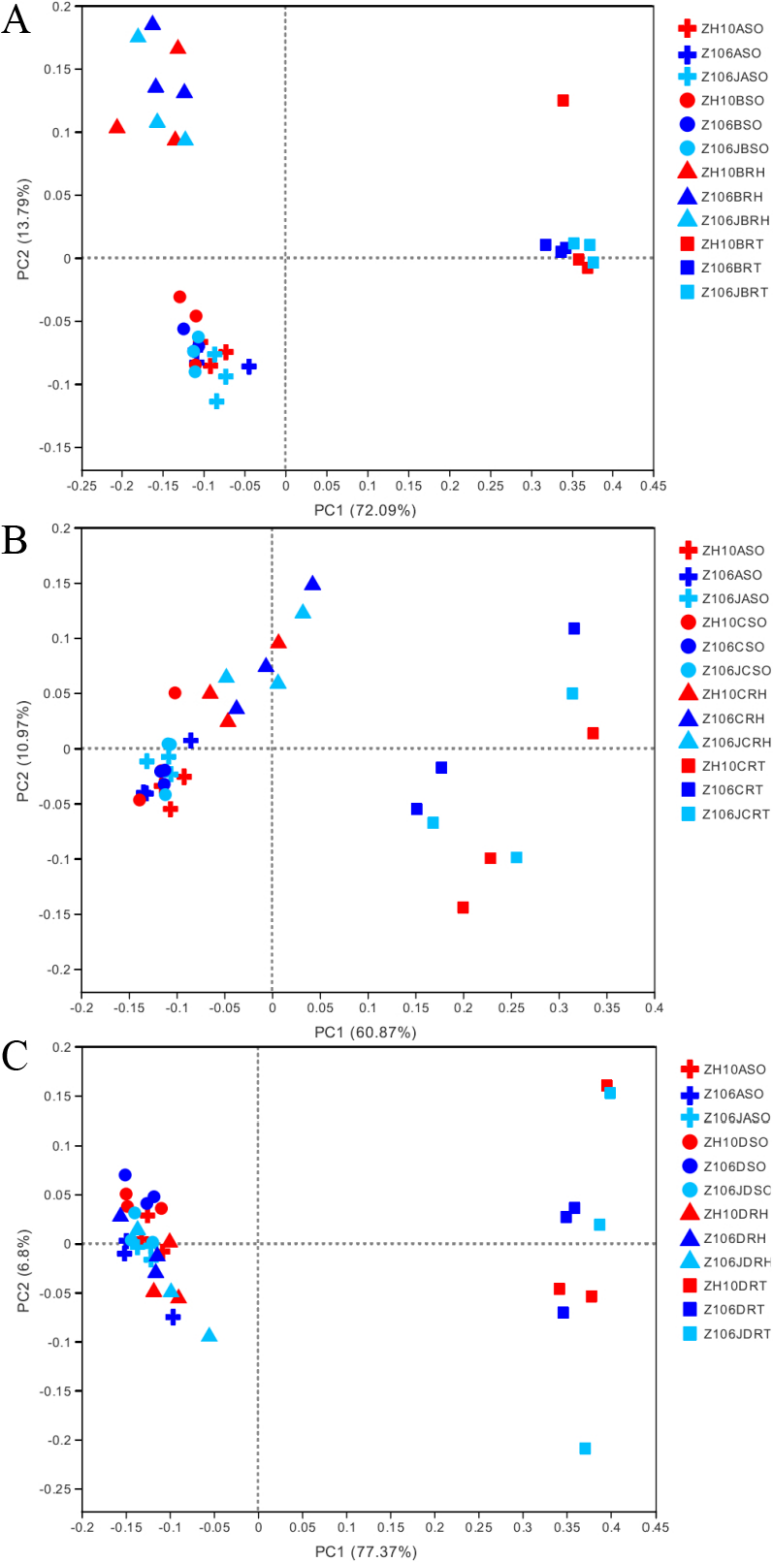
PCoA based on the weighted UF distance matrix at three stages among three treatments. A) Seedling; B) flowering; C) seed filling. Z106 and ZH10 represent the transgenic soybean (ZH10-6) and its recipient cultivar (Zhonghuang 10), respectively. Z106J represents Z106 treated with glyphosate. A represents soil before planting, B, C, and D represent the seedling, flowering, and seed filling stages, respectively. SO, RH, and RT represent the surrounding soil, rhizospheric soil, and root samples, respectively.

The normalized abundance of each OTU in each sample was calculated based on the OTU table for the biom format, and a beta diversity analysis was performed by PCoA based on the WUF distance (Fig. 2) of the root-associated bacterial communities of ZH10, Z106, and Z106J. The distance between samples was assessed by composition similarities among all soil samples. As shown in Fig. 2, bacterial communities in the surrounding soil, rhizospheric soil, and root samples of ZH10, Z106, and Z106J were not distinct from each other at the seedling, flowering, or seed filling stages, indicating no marked difference among the three treatments. In addition, the bacterial communities in the root samples of ZH10, Z106 and Z106J were distinct from those in the surrounding soil and rhizospheric soil samples at all three stages. The results of PLS-DA were also consistent with the above results (Fig. S5).

We then performed statistical ANOSIM and Adonis on bacterial communities. The results of ANOSIM and Adonis based on the Bray–Curtis distance both indicated that the taxonomic beta diversities of bacterial communities among ZH10, Z106, and Z106J in the same compartment at one stage were not significantly different (Table S3).

In order to verify the results obtained, we performed ANOSIM and Adonis based on WUF and UUF (Table S3), and these results also indicated no significant differences among the phylogenetic beta diversity of bacterial communities among ZH10, Z106, and Z106J in the same compartment at different stages.

### Comparative analysis of taxonomic levels of root-associated bacterial communities among ZH10, Z106, and Z106J

**Fig. 3.**
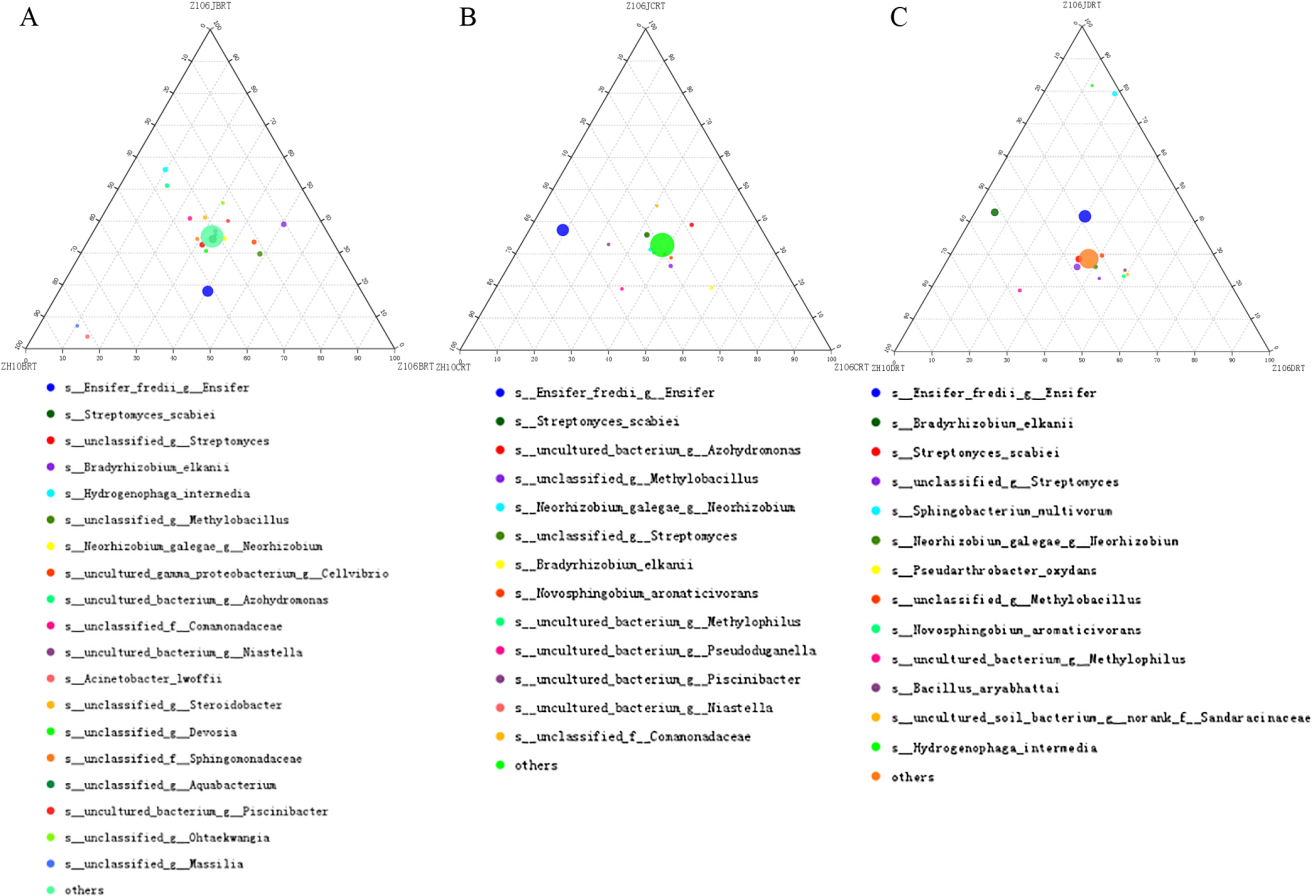
Ternary plot of species based on different treatments in root samples at three stages. Different proportional contributions were observed: A) Seedling; B) flowering; C) seed filling. Z106 and ZH10 represent the transgenic soybean (ZH10-6) and its recipient cultivar (Zhonghuang 10), respectively. Z106J represents Z106 treated with glyphosate. B, C, and D represent the seedling, flowering, and seed filling stages, respectively. RT represents the root sample.

We used a ternary plot analysis to identify the bacteria responsible for the observed community differentiation at the species level and the results obtained showed no marked differences among the three treatments in the surrounding soil or rhizospheric soil samples; however, several species had higher proportions in root samples (Fig. S7C), including *Ensifer* (previously described as *Sinorhizobium*) *fredii* belonging to the family *Rhizobiaceae*, order *Rhizobiales*, and class *Alphaproteobacteria* within the phylum *Proteobacteria*. In comparisons with Z106, *E. fredii* accounted for a lower proportional contribution in Z106J at the seedling stage (Fig. S7D) and a higher proportional contribution in ZH10 and Z106J at the flowering stage (Fig. S7E). In the seed filling stage, the proportional contribution of *E. fredii* among the three treatments appeared to be similar (Fig. S7F). Based on these results, we conducted the same analysis of root samples at the three stages among the three treatments, and the results obtained were consistent with the ternary plots shown in Fig. S7D, E, and F (Fig. 3). Regarding the species *Bradyrhizobium elkanii*, which belongs to the family *Bradyrhizobiaceae*, order *Rhizobiales*, and class *Alphaproteobacteria* within the phylum *Proteobacteria*, its proportional contribution in Z106 was higher than that in ZH10 and Z106J at the seedling and flowering stages, but was lower at the seed filling stage.

We also compared the relative abundance of major five bacterial taxa at the phylum, class, order, family, genus, and species taxonomic levels using a one-way ANOVA (Fig. S8). The results revealed that the relative abundance of *Betaproteobacteria* (Fig. S8D), *Burkholderiales* (Fig. S8G) and *Comamonadaceae* (Fig. S8J) in root samples at the seedling stage was significantly higher in Z106J than in Z106, while the relative abundance of *Burkholderiales* (Fig. S8G) and *Comamonadaceae* (Fig. S8J) was significantly higher in ZH10 than in Z106. Regarding root samples at the flowering stage, the relative abundance of *Alphaproteobacteria* (Fig. S8E), *Rhizobiales* (Fig. S8H), and *Rhizobiaceae* (Fig. S8K) was significantly higher in ZH10 than in Z106.

### Comparison of the composition of main nitrogen-fixing bacterial genera at different growth stages

**Fig. 4.**
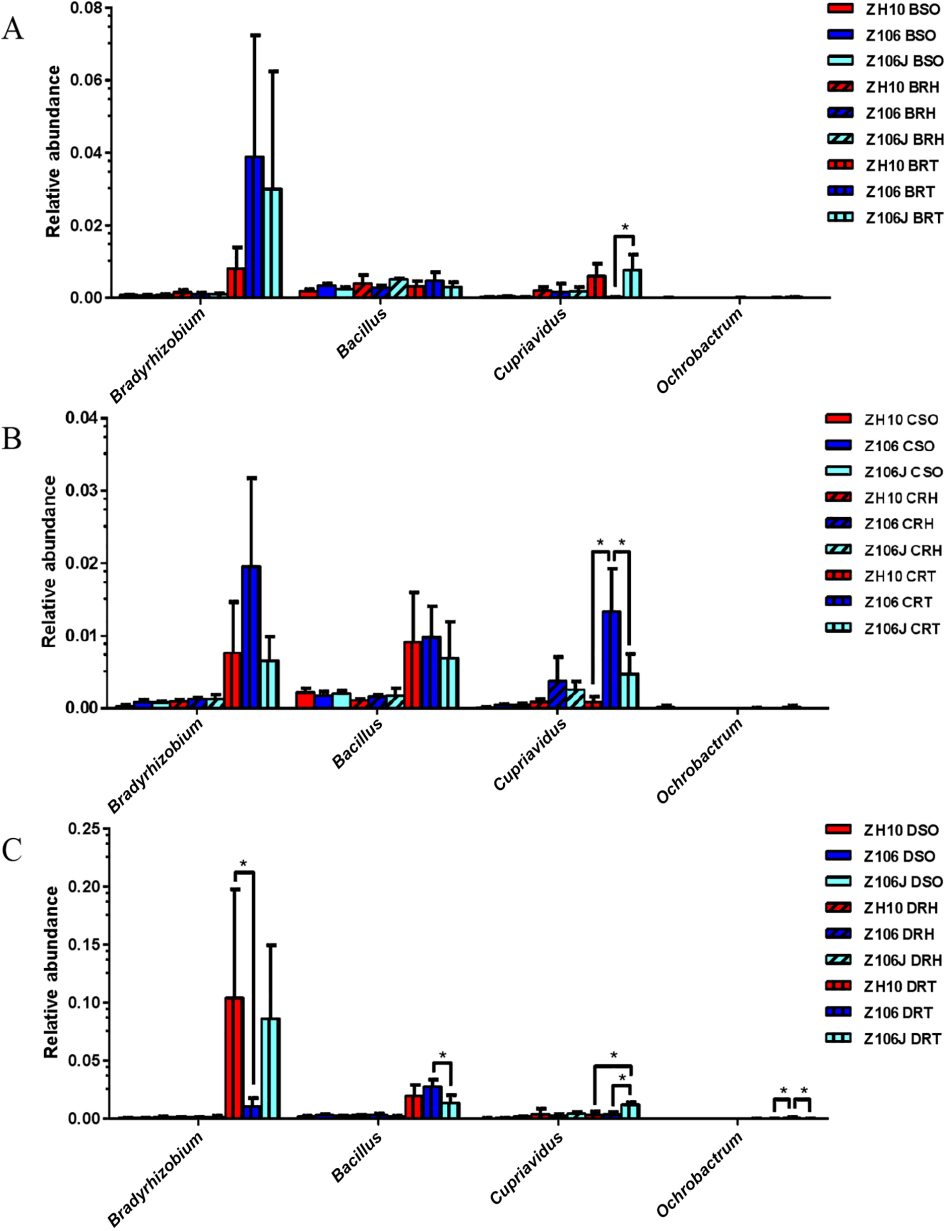
Relative abundance of main nitrogen-fixing bacterial genera among three treatments at three stages. A) Seedling; B) flowering; C) seed filling. Z106 and ZH10 represent the transgenic soybean (ZH10-6) and its recipient cultivar (Zhonghuang 10), respectively. Z106J represents Z106 treated with glyphosate. B, C, and D represent the seedling, flowering, and seed filling stages, respectively. SO, RH, and RT represent the surrounding soil, rhizospheric soil, and root samples, respectively. The asterisk (*, *P*<0.05) indicates a significant difference according to a one-way ANOVA.

We examined the relative abundance of the main nitrogen-fixing bacterial genera and found that the relative abundance of *Cupriavidus* was significantly higher in the Z106J root sample than in the Z106 root sample at the seedling and seed filling stages (Fig. 4A and C). At the flowering stage, the relative abundance of *Cupriavidus* was significantly higher in the Z106 root sample than in the ZH10 and Z106J root samples (Fig. 4B). Regarding root samples at the seed filling stage, the relative abundance of *Bradyrhizobium* was significantly higher in ZH10 than in Z106, while the relative abundance of *Bacillus* and *Ochrobactrum* was significantly higher in Z106 than in Z106J, and *Ochrobactrum* was present at a significantly higher abundance in Z106 than in ZH10 (Fig. 4C).

## Discussion

The general concept of the present study was partly derived from previous studies (Mendes *et al.*, 2014; Edwards *et al.*, 2015) that examined the microbial communities associated with three rhizo-compartments. In the present study, we used similar compartments, including the surrounding soil, rhizospheric soil and associated roots containing the rhizoplane and endosphere (Bulgarelli *et al.*, 2012; Edwards *et al.*, 2015; Lu *et al.*, 2018b; Wen *et al.*, 2019), to effectively compare differences among the three soybean treatments at the three growth stages and assess the impact of GM crops on underground microbial communities.

In the present study, we selected the glyphosate-resistant soybean line Z106 with the dual transgenes of *G2-EPSPS* and *GAT* and its recipient cultivar ZH10 as research materials. In contrast to conventional comparisons, which generally focus on the effects of transgenic or only herbicide application separately (Babujia *et al.*, 2016; Fan *et al.*, 2017), we evaluated the influence of glyphosate-resistant transgenes together with the application of glyphosate. Thus, the impact of the dual transgenic soybean between Z106 and ZH10 and that of glyphosate between Z106 treated with glyphosate (Z106J) and Z106 with clean water on soil root-associated microbial communities were effectively analyzed at the seedling, flowering, and seed filling stages, which appeared to clearly provide an important reference for the evaluation of soil ecological and environmental safety (Bulgarelli *et al.*, 2012).

In the alpha diversity analysis, no significant differences were observed in the surrounding soil, rhizospheric soil, or root samples of ZH10, Z106, and Z106J at the seedling, flowering, or seed filling stages (Table S2). Similarly, the beta diversity analysis failed to detect significant differences among the three treatments in the same compartment at any of the three stages (Table S3). These results indicate that neither the *G2-EPSPS & GAT* transgenes nor the application of glyphosate significantly changed the community structure of soil microbial communities.

However, in comparisons of the composition of the major bacterial taxa at the three growth stages, the abundance of some soil bacterial communities showed significant differences among the three treatments (Fig. S8). Ternary plots also revealed differences in the proportional contributions of several root-associated microbial communities based on different compartments, treatments, or stages. As shown in Fig. 3, the proportional contribution of *E. fredii* was higher in root samples at the flowering stage of ZH10 and Z106J than that of Z106, which is reported to be involved in nitrogen-fixing nodule formation in plants and is a very common symbiont of soybean (Temprano-Vera *et al.*, 2018) and other legumes (Rathi *et al.*, 2018) in alkaline soils, in which it forms determinate nodules in soybean and cowpea (Krishnan, 2002; Lopez-Baena *et al.*, 2016). Furthermore, *E. fredii* is a fast-growing root nodule bacterium with a broad spectrum of hosts that was initially isolated from Chinese soil and nodulates the roots of 79 diverse genera of legumes (Krishnan, 2002; Albareda *et al.*, 2009).

The lower proportional contribution of *E. fredii* in Z106 than in ZH10 indicated that the glyphosate-resistant transgenes *G2-EPSPS & GAT* affected root-associated microbial diversity in the root system of soybean, possibly through changes in gene expression and root exudation, as reported previously in GM crops (Tesfaye *et al.*, 2003; Steinauer *et al.*, 2016). Compared to Z106, the exogenous application of glyphosate recovered the proportional contribution of *E. fredii* in Z106J, which was similar to that of ZH10. A previous study reported that a novel class II *EPSPS* from *E. fredii* showed a high level of glyphosate tolerance (Wang *et al.*, 2014), which reflected the potential ability of *E. fredii* to resist glyphosate, suggesting positive feedback through which glyphosate resistance occurs when Z106 is treated with glyphosate. *GAT* may also degrade glyphosate, which then acts as a nutrient source for *E. fredii*, thereby neutralizing its herbicidal effects (Saxton *et al.*, 2011).

The relative abundance of nitrogen-fixing bacterial genera in soil was also investigated in the present study, and the results obtained showed that the relative abundance of *Bradyrhizobium* was significantly influenced by the *G2-EPSPS & GAT* transgenes at the seed filling stage (Fig. 4). The genus *Bradyrhizobium* is generally reported to be related to soybean nitrogen fixation, such as *B. elkanii*, which is a nitrogen-fixing bacterium that nodulates soybean (Barcellos *et al.*, 2007; de Souza *et al.*, 2012; Mason *et al.*, 2018). In addition, *B. elkanii* showed different proportional contributions among the three treatments at the three stages (Fig. 3), which also demonstrated that the *G2-EPSPS & GAT* transgenes and the application of glyphosate may affect the abundance of nitrogen-fixing bacteria. Notably, the higher proportional contribution of *B. elkanii* in ZH10 and Z106J at the seed filling stage was similar to the proportional contribution of the nitrogen-fixing species *E. fredii* among the three treatments at the flowering stage.

In the present study, differences in relative abundance among the three treatments may also be partly due to variations in soil pH (Table 1) caused by transgenic soybean planting or the application of glyphosate. Soil pH is generally regarded as one of the major factors influencing microbial activity or the community structure (Aciego Pietri and Brookes, 2009; Cao *et al.*, 2016); however, this needs to be examined in more detail in order to elucidate the mechanisms responsible for the different responses.

To clarify the mechanisms by which the crop affected the rhizosphere diazotrophic community, the relative abundance of nitrogen-fixing bacteria in root nodule samples and biological nitrogen fixation (BNF) by soybean need to be assessed (Bohm *et al.*, 2009; Hungria *et al.*, 2014; Fan *et al.*, 2017); however, in the present study, no significant differences were observed in the number of nodules among the three treatments (data not shown). This may be due to the sampling error of nodules, which ultimately resulted in the cessation of attempts to extract bacterial genomic DNA from nodules. However, this issue needs to be examined in future studies to fully elucidate the relative abundance and responses of nitrogen-fixing bacteria residing not only in root-associated compartments, but also in root nodules.

*G2*-*EPSPS & GAT* transgenic herbicide-tolerant soybean treated with or without glyphosate had no significant impact on rhizosphere microbial communities in soil during a single growth season. However, *G2-EPSPS & GAT* transgenes and the application of glyphosate specifically affected the soil microbial community structure, such as the relative abundance and proportional contributions of some nitrogen-fixing bacteria in soybean root-associated bacterial communities, which may have an impact on rhizosphere nitrogen fixation efficiency in soybean, particularly those used for soil remediation under abiotic stresses. For example, in acidic soils lacking efficient phosphorus with aluminum toxicity (Barcelo and Poschenrieder, 2002; Zhao *et al.*, 2004; Liao *et al.*, 2006), soybean plants with nitrogen fixation ability were planted with the expectation of soil improvement (Cheng *et al.*, 2009; Yang *et al.*, 2012); therefore, there are concerns about the efficiency of soil remediation, in addition to assessment of biosafety issues, if application of these GM soybeans is expanded. In addition, the present results need to be confirmed in studies using more biological replicates and field experiments under different geographical and physiological conditions.

## Citation

Yang, M., Wen, Z., Fazal, A., Hua, X., Xu, X., Yin, T., et al. (2020) Impact of a *G2-EPSPS* & *GAT* Dual Transgenic Glyphosate-Resistant Soybean Line on the Soil Microbial Community under Field Conditions Affected by Glyphosate Application. *Microbes Environ ***35**: ME20056.

https://doi.org/10.1264/jsme2.ME20056

## Supplementary Material

Supplementary Material

## Figures and Tables

**Table 1.** Surrounding soil and plant analyses of ZH10, Z106, and Z106J showed significant differences in some traits among three treatments.

**Analysis**	**Trait**	**Seedling Stage (Mean±SD)**				**Flowering Stage (Mean±SD)**				**Seed Filling Stage (Mean±SD)**		
		**ZH10**	**Z106**	**Z106J**		**ZH10**	**Z106**	**Z106J**		**ZH10**	**Z106**	**Z106J**
**Soil analysis**	**pH value**	**8.12±0.04^a^**	**7.79±0.14^b^**	**7.82±0.05^b^**		**7.95±0.04^b^**	**8.01±0.03^b^**	**8.11±0.03^a^**		**7.92±0.04^b^**	**8.00±0.02^a^**	**7.96±0.01^b^**
	**Water content (%)**	**10.85±0.01^a^**	**12.67±0.01^b^**	**11.53±0.01^ab^**		**14.65±0.00^b^**	**15.54±0.00^a^**	**14.70±0.00^b^**		**10.58±0.01^b^**	**10.98±0.01^b^**	**11.48±0.00^a^**
	**C content (%)**	0.95±0.05	1.00±0.03	1.00±0.05		1.03±0.10	0.96±0.06	0.97±0.06		**0.99±0.03^b^**	**1.12±0.02^a^**	**0.97±0.06^b^**
	**N content (%)**	0.10±0.00	0.11±0.01	0.10±0.01		0.10±0.01	0.10±0.01	0.10±0.01		0.10±0.01	0.10±0.01	0.10±0.01
	Ethylene production (nmol [g*h]^–1^)	**14.91±4.90^a^**	**22.53±2.28^b^**	**23.64±9.69^b^**		56.64±23.71	63.53±14.98	52.33±14.02		**21.06±5.11^a^**	**25.30±4.89^ab^**	**32.27±5.25^b^**
**Plant analysis**	**C content (%)**	**38.38±0.20^a^**	**39.91±0.41^b^**	**39.76±0.63^b^**		**40.17±0.81^b^**	**37.75±1.15^a^**	**39.38±0.55^b^**		38.79±1.36	38.88±0.56	39.51±0.57
	**N content (%)**	**4.03±0.11^a^**	**4.41±0.16^b^**	**4.62±0.11^c^**		**2.96±0.17^a^**	**3.37±0.13^b^**	**3.40±0.15^b^**		**2.34±0.18^a^**	**2.56±0.26^ab^**	**2.85±0.10^b^**

SD represents the standard deviation (*n*=3). Z106 and ZH10 represent transgenic soybean ZH10-6 and its recipient cultivar Zhonghuang 10. Z106J represents Z106 treated with glyphosate. C and N contents represent carbon and nitrogen contents, respectively. Statistical analyses were performed using a one-way ANOVA. The values in bold indicate a significant difference (*P*<0.05) among the ZH10, Z106, and Z106J groups.

## References

[B1] Aciego Pietri J.C., and Brookes P.C. (2009) Substrate inputs and pH as factors controlling microbial biomass, activity and community structure in an arable soil. Soil Biol Biochem 41: 1396–1405.

[B2] Albareda M., Rodríguez-Navarro D.N., and Temprano F.J. (2009) Use of *Sinorhizobium (Ensifer) fredii* for soybean inoculants in South Spain. Eur J Agron 30: 205–211.

[B3] Andam C.P., Mondo S.J., and Parker M.A. (2007) Monophyly of *nodA* and *nifH* genes across texan and costa rican populations of *Cupriavidus* nodule symbionts. Appl Environ Microbiol 73: 4686–4690. 17526782 10.1128/AEM.00160-07 PMC1932833

[B4] Babujia L.C., Silva A.P., Nakatani A.S., Cantão M.E., Vasconcelos A.T.R., Visentainer J.V., and Hungria M. (2016) Impact of long-term cropping of glyphosate-resistant transgenic soybean [*Glycine max* (L.) Merr.] on soil microbiome. Transgenic Res 25: 425–440. 26873023 10.1007/s11248-016-9938-4

[B5] Barcellos F.G., Menna P., Batista J.S.D., and Hungria M. (2007) Evidence of horizontal transfer of symbiotic genes from a *Bradyrhizobium japonicum* inoculant strain to indigenous diazotrophs *Sinorhizobium (Ensifer) fredii* and *Bradyrhizobium elkanii* in a Brazilian Savannah soil. Appl Environ Microbiol 73: 2635–2643. 17308185 10.1128/AEM.01823-06 PMC1855619

[B6] Barcelo J., and Poschenrieder C. (2002) Fast root growth responses, root exudates, and internal detoxification as clues to the mechanisms of aluminium toxicity and resistance: a review. Environ Exp Bot 48: 75–92.

[B7] Benbrook C.M. (2016) Trends in glyphosate herbicide use in the United States and globally. Environ Sci Eur 28: 3. 27752438 10.1186/s12302-016-0070-0 PMC5044953

[B8] Bohm G.M.B., Alves B.J.R., Urquiaga S., Boddey R.M., Xavier G.R., Hax F., and Rombaldi C.V. (2009) Glyphosate- and imazethapyr-induced effects on yield, nodule mass and biological nitrogen fixation in field-grown glyphosate-resistant soybean. Soil Biol Biochem 41: 420–422.

[B9] Brookes G., and Barfoot P. (2013) The global income and production effects of genetically modified (GM) crops 1996–2011. GM Crops Food 4: 74–83. 23549349 10.4161/gmcr.24176

[B10] Bulgarelli D., Rott M., Schlaeppi K., van Themaat E.V.L., Ahmadinejad N., Assenza F., et al. (2012) Revealing structure and assembly cues for *Arabidopsis* root-inhabiting bacterial microbiota. Nature 488: 91–95. 22859207 10.1038/nature11336

[B11] Cao H.C., Chen R.R., Wang L.B., Jiang L.L., Yang F., Zheng S.X., et al. (2016) Soil pH, total phosphorus, climate and distance are the major factors influencing microbial activity at a regional spatial scale. Sci Rep 6: 25815. 27170469 10.1038/srep25815 PMC4864422

[B12] Caporaso J.G., Kuczynski J., Stombaugh J., Bittinger K., Bushman F.D., Costello E.K., et al. (2010) QIIME allows analysis of high-throughput community sequencing data. Nat Methods 7: 335–336. 20383131 10.1038/nmeth.f.303 PMC3156573

[B13] Castle L.A., Siehl D.L., Gorton R., Patten P.A., Chen Y.H., Bertain S., et al. (2004) Discovery and directed evolution of a glyphosate tolerance gene. Science 304: 1151–1154. 15155947 10.1126/science.1096770

[B14] Cheng F.X., Cao G.Q., Wang X.R., Zhao J., Yan X.L., and Liao H. (2009) Isolation and application of effective nitrogen fixation rhizobial strains on low-phosphorus acid soils in South China. Chin Sci Bull 54: 412–420.

[B15] Dale P.J., Clarke B., and Fontes E.M.G. (2002) Potential for the environmental impact of transgenic crops. Nat Biotechnol 20: 567–574. 12042859 10.1038/nbt0602-567

[B16] de Souza J.A.M., Tieppo E., Magnani G.D.S., Alves L.M.C., Cardoso R.L., Cruz L.M., et al. (2012) Draft Genome sequence of the nitrogen-fixing symbiotic bacterium *Bradyrhizobium elkanii* 587. J Bacteriol 194: 3547–3548. 22689236 10.1128/JB.00563-12 PMC3434731

[B17] Duke S.O., and Powles S.B. (2008) Glyphosate: a once-in-a-century herbicide. Pest Manage Sci 64: 319–325. 10.1002/ps.1518 18273882

[B18] Edgar R.C., Haas B.J., Clemente J.C., Quince C., and Knight R. (2011) UCHIME improves sensitivity and speed of chimera detection. Bioinformatics 27: 2194–2200. 21700674 10.1093/bioinformatics/btr381 PMC3150044

[B19] Edgar R.C. (2013) UPARSE: highly accurate OTU sequences from microbial amplicon reads. Nat Methods 10: 996–998. 23955772 10.1038/nmeth.2604

[B20] Edwards J., Johnson C., Santos-Medellín C., Lurie E., Podishetty N.K., Bhatnagar S., et al. (2015) Structure, variation, and assembly of the root-associated microbiomes of rice. Proc Natl Acad Sci U S A 112: E911–E920. 25605935 10.1073/pnas.1414592112 PMC4345613

[B21] Fadrosh D.W., Ma B., Gajer P., Sengamalay N., Ott S., Brotman R.M., and Ravel J. (2014) An improved dual-indexing approach for multiplexed 16S rRNA gene sequencing on the Illumina MiSeq platform. Microbiome 2: 6. 24558975 10.1186/2049-2618-2-6 PMC3940169

[B22] Fan L., Feng Y.C., Weaver D.B., Delaney D.P., Wehtje G.R., and Wang G.Y. (2017) Glyphosate effects on symbiotic nitrogen fixation in glyphosate-resistant soybean. Appl Soil Ecol 121: 11–19.

[B23] Feng J., and Yang F. (2019) The regulation of genetically modified food in China. Biotechnol Law Rep 38: 289–293.

[B24] Guo B.F., Guo Y., Hong H.L., Jin L.G., Zhang L.J., Chang R.Z., et al. (2015) Co-expression of *G2-EPSPS* and glyphosate acetyltransferase *GAT* genes conferring high tolerance to glyphosate in soybean. Front Plant Sci 6: 847. 26528311 10.3389/fpls.2015.00847 PMC4606067

[B25] Guo B.F., Guo Y., Hong H.L., and Qiu L.J. (2016) Identification of genomic insertion and flanking sequence of *G2-EPSPS* and *GAT* transgenes in soybean using whole genome sequencing method. Front Plant Sci 7: 1009. 27462336 10.3389/fpls.2016.01009 PMC4940375

[B26] Hungria M., Mendes I.C., Nakatani A.S., dos Reis F.B., Morais J.Z., de Oliveira M.C.N., and Fernandes M.F. (2014) Effects of the glyphosate-resistance gene and herbicides on soybean: Field trials monitoring biological nitrogen fixation and yield. Field Crops Res 158: 43–54.

[B27] ISAAA (2017) Global Status of Commercialized Biotech/GM Crops: 2016. New York, NY: The International Service for the Acquisition of Agri-biotech Applications (ISAAA).

[B28] ISAAA (2018) Global Status of Commercialized Biotech/GM Crops in 2017: Biotech Crop Adoption Surges as Economic Benefits Accumulate in 22 Years. New York, NY: The International Service for the Acquisition of Agri-biotech Applications (ISAAA).

[B29] Jie W.G., Lin J.X., Guo N., Cai B.Y., and Yan X.F. (2019) Community composition of rhizosphere fungi as affected by *Funneliformis mosseae* in soybean continuous cropping soil during seedling period. Chil J Agric Res 79: 356–365.

[B30] Kaneko T., Nakamura Y., Sato S., Minamisawa K., Uchiumi T., Sasamoto S., et al. (2002) Complete genomic sequence of nitrogen-fixing symbiotic bacterium *Bradyrhizobium japonicum* USDA110. DNA Res 9: 189–197. 12597275 10.1093/dnares/9.6.189

[B31] Krishnan H.B. (2002) NolX of *Sinorhizobium fredii* USDA257, a type III-secreted protein involved in host range determination, Iis localized in the infection threads of cowpea (*Vigna unguiculata* [L.] Walp) and soybean (*Glycine max* [L.] Merr.) nodules. J Bacteriol 184: 831–839. 11790754 10.1128/JB.184.3.831-839.2002 PMC139521

[B32] Liao H., Wan H., Shaff J., Wang X., Yan X., and Kochian L.V. (2006) Phosphorus and aluminum interactions in soybean in relation to aluminum tolerance. Exudation of specific organic acids from different regions of the intact root system. Plant Physiol 141: 674–684. 16648222 10.1104/pp.105.076497 PMC1475464

[B33] Liu X., Wei S., Wang F., James E.K., Guo X., Zagar C., et al. (2012) *Burkholderia* and *Cupriavidus* spp. are the preferred symbionts of *Mimosa* spp. in southern China. FEMS Microbiol Ecol 80: 417–426. 22268711 10.1111/j.1574-6941.2012.01310.x

[B34] Lopez-Baena F.J., Ruiz-Sainz J.E., Rodriguez-Carvajal M.A., and Vinardell J.M. (2016) Bacterial molecular signals in the *Sinorhizobium fredii*-soybean symbiosis. Int J Mol Sci 17: 755. 10.3390/ijms17050755 PMC4881576 27213334

[B35] Lu G.H., Zhu Y.L., Kong L.R., Cheng J., Tang C.Y., Hua X.M., et al. (2017) Impact of a glyphosate-tolerant soybean line on the rhizobacteria, revealed by Illumina MiSeq. J Microbiol Biotechnol 27: 561–572. 27974727 10.4014/jmb.1609.09008

[B36] Lu G.H., Hua X.M., Cheng J., Zhu Y.L., Wang G.H., Pang Y.J., et al. (2018a) Impact of glyphosate on the rhizosphere microbial communities of an *EPSPS*-transgenic soybean line ZUTS31 by metagenome sequencing. Curr Genomics 19: 36–49. 29491731 10.2174/1389202918666170705162405 PMC5817875

[B37] Lu G.H., Tang C.Y., Hua X.M., Cheng J., Wang G.H., Zhu Y.L., et al. (2018b) Effects of an *EPSPS*-transgenic soybean line ZUTS31 on root-associated bacterial communities during field growth. PLoS One 13: e0192008. 29408918 10.1371/journal.pone.0192008 PMC5800644

[B38] Mason M.L.T., Tabing B.L.C., Yamamoto A., and Saeki Y. (2018) Influence of flooding and soil properties on the genetic diversity and distribution of indigenous soybean-nodulating bradyrhizobia in the Philippines. Heliyon 4: e00921. 30480155 10.1016/j.heliyon.2018.e00921 PMC6240709

[B39] Mendes L.W., Kuramae E.E., Navarrete A.A., van Veen J.A., and Tsai S.M. (2014) Taxonomical and functional microbial community selection in soybean rhizosphere. ISME J 8: 1577–1587. 24553468 10.1038/ismej.2014.17 PMC4817605

[B40] Ngom A., Nakagawa Y., Sawada H., Tsukahara J., Wakabayashi S., Uchiumi T., et al. (2004) A novel symbiotic nitrogen-fixing member of the *Ochrobactrum* clade isolated from root nodules of *Acacia mangium*. J Gen Appl Microbiol 50: 17–27. 15057707 10.2323/jgam.50.17

[B41] Peiffer J.A., Spor A., Koren O., Jin Z., Tringe S.G., Dangl J.L., et al. (2013) Diversity and heritability of the maize rhizosphere microbiome under field conditions. Proc Natl Acad Sci U S A 110: 6548–6553. 23576752 10.1073/pnas.1302837110 PMC3631645

[B42] Rathi S., Tak N., Bissa G., Chouhan B., Ojha A., Adhikari D., et al. (2018) Selection of *Bradyrhizobium* or *Ensifer* symbionts by the native Indian caesalpinioid legume *Chamaecrista pumila* depends on soil pH and other edaphic and climatic factors. FEMS Microbiol Ecol 94: fiy180. 10.1093/femsec/fiy180 30184201

[B43] Rohart F., Gautier B., Singh A., and Lê Cao K.A. (2017) mixOmics: An R package for ‘omics feature selection and multiple data integration. PLoS Comput Biol 13: e1005752. 29099853 10.1371/journal.pcbi.1005752 PMC5687754

[B44] Saxton M.A., Morrow E.A., Bourbonniere R.A., and Wilhelm S.W. (2011) Glyphosate influence on phytoplankton community structure in Lake Erie. J Great Lakes Res 37: 683–690.

[B45] Schlaeppi K., Dombrowski N., Oter R.G., Ver Loren van Themaat E., and Schulze-Lefert P. (2014) Quantitative divergence of the bacterial root microbiota in *Arabidopsis thaliana* relatives. Proc Natl Acad Sci U S A 111: 585–592. 24379374 10.1073/pnas.1321597111 PMC3896156

[B46] Schloss P.D., Westcott S.L., Ryabin T., Hall J.R., Hartmann M., Hollister E.B., et al. (2009) Introducing mothur: open-source, platform-independent, community-supported software for describing and comparing microbial communities. Appl Environ Microbiol 75: 7537–7541. 19801464 10.1128/AEM.01541-09 PMC2786419

[B47] Seldin L., and Dubnau D. (1985) Deoxyribonucleic-acid homology among *Bacillus-polymyxa*, *Bacillus-macerans*, *Bacillusazotofixans*, and other nitrogen-fixing *Bacillus* strains. Int J Syst Bacteriol 35: 151–154.

[B48] Steinauer K., Chatzinotas A., and Eisenhauer N. (2016) Root exudate cocktails: the link between plant diversity and soil microorganisms? Ecol Evol 6: 7387–7396. 28725406 10.1002/ece3.2454 PMC5513276

[B49] Talebi M., Emtiazi G., Sepahy A.A., and Zaghian S. (2013) Zymogram analysis of alkaline keratinase produced by nitrogen fixing *Bacillus pumilus* ZED17 exhibiting multiprotease enzyme activities. Jundishapur J Microbiol 6: e7974.

[B50] Temprano-Vera F., Rodríguez-Navarro D.N., Acosta-Jurado S., Perret X., Fossou R.K., Navarro-Gomez P., et al. (2018) *Sinorhizobium fredii* strains HH103 and NGR234 form nitrogen fixing nodules with diverse wild soybeans (*Glycine soja*) from central China but are ineffective on Northern China accessions. Front Microbiol 9: 2843. 30519234 10.3389/fmicb.2018.02843 PMC6258812

[B51] Tesfaye M., Dufault N.S., Dornbusch M.R., Allan D.L., Vance C.P., and Samac D.A. (2003) Influence of enhanced malate dehydrogenase expression by alfalfa on diversity of rhizobacteria and soil nutrient availability. Soil Biol Biochem 35: 1103–1113.

[B52] Tian X.L., Wang C.B., Bao X.G., Wang P., Li X.F., Yang S.C., et al. (2019) Crop diversity facilitates soil aggregation in relation to soil microbial community composition driven by intercropping. Plant Soil 436: 173–192.

[B53] Wackerow-Kouzova N.D. (2007) *Ochrobactrum intermedium* ANKI, a nitrogen-fixing bacterium able to decolorize azobenzene. Appl Biochem Microbiol 43: 403–406. 17929573

[B54] Wang L., Peng R., Tian Y., Han J., Zhao W., Wang B., et al. (2014) Characterization of a class II 5-enopyruvylshikimate-3-phosphate synthase with high tolerance to glyphosate from *Sinorhizobium fredii*. World J Microbiol Biotechnol 30: 2967–2973. 25151150 10.1007/s11274-014-1724-y

[B55] Wen Z.L., Yang M.K., Du M.H., Zhong Z.Z., Lu Y.T., Wang G.H., et al. (2019) Enrichments/derichments of root-associated bacteria related to plant growth and nutrition caused by the growth of an *EPSPS*-transgenic maize line in the field. Front Microbiol 10: 1335. 31275269 10.3389/fmicb.2019.01335 PMC6591461

[B56] Yang T.Y., Liu G.L., Li Y.C., Zhu S.M., Zou A.L., Qi J.L., and Yang Y.H. (2012) Rhizosphere microbial communities and organic acids secreted by aluminum-tolerant and aluminum-sensitive soybean in acid soil. Biol Fertil Soils 48: 97–108.

[B57] Zhang J., Zhang B., Liu Y., Guo Y., Shi P., and Wei G. (2018) Distinct large-scale biogeographic patterns of fungal communities in bulk soil and soybean rhizosphere in China. Sci Total Environ 644: 791–800. 29990927 10.1016/j.scitotenv.2018.07.016

[B58] Zhao J., Fu J.B., Liao H., He Y., Nian H., Hu Y. M., et al. (2004) Characterization of root architecture in an applied core collection for phosphorus efficiency of soybean germplasm. Chin Sci Bull 49: 1611–1620.

