# Supplementary material for: Impact of a G2-EPSPS & GAT Dual Transgenic Glyphosate-Resistant Soybean Line on the Soil Microbial Community under Field Conditions Affected by Glyphosate Application
Source: Microbes Environ. 2020 Nov 7;35(4):ME20056. doi: 10.1264/jsme2.ME20056 (PMC7734404; doi:10.1264/jsme2.ME20056)
Supplement: Supplementary file 1 — Supplementary Material [file 35_20056_s1.pdf]

Supplementary Materials

Table S1: Summary of sequencing data of all samples

Table S2: *P*-values of six indices for alpha diversity of the different compartment samples between ZH10, Z106, and Z106J according to Student's *t*-test

Table S3: ANOSIM and Adonis of the different compartment samples between ZH10, Z106, and Z106J based on the Bray–Curtis, Weighted Unifrac and Unweighted Unifrac distance metrics

Figure S1: Field design and sampling sites

Figure S2: Rank-Abundance curves of all 90 samples

Figure S3: Pan-Core analysis of all samples

Figure S4: The remaining boxplots of alpha diversity

Figure S5: Partial Least Squares Discriminant Analysis on OTU level at seedling, flowering, and seed-filling stages

Figure S6: Community barplot analysis of all samples

Figure S7: Ternary plot of species based on different treatments in surrounding soil, rhizospheric soil, root, seedling stage, flowering stage and seed-filling stage

Figure S8: The relative abundance scatter plot of the major five bacterial taxa among three treatments in different stages at Phylum (A, B, C), Class (D, E, F), Order (G, H, I), Family (J, K, L), Genus (M, N, O) and Species (P, Q, R) level.

Table S1: Summary of sequencing data of all samples

Sample Name	Reads Length (bp)	Raw Data (Mbp)	Adapter (%)	N base (%)	Ploy base (%)	Low Quality (%)	Clean Data (Mbp)	Data Utilization Ratio (%)	Raw Reads	Clean Reads	Read Utilization Ratio (%)
Z106ASO12	250:250	66.81	0.000	0.000	0.018	3.179	62.52	93.59	133611*2	125804*2	94.16
Z106ASO34	250:250	70.44	0.000	0.000	0.025	3.043	66.13	93.89	140883*2	132957*2	94.37
Z106ASO56	250:250	85.53	0.000	0.000	0.018	2.993	80.39	93.99	171055*2	161581*2	94.46
Z106BRH12	250:250	52.80	0.000	0.000	0.026	3.027	49.58	93.90	105594*2	99609*2	94.33
Z106BRH34	250:250	54.06	0.000	0.000	0.025	2.839	50.96	94.28	108119*2	102417*2	94.73
Z106BRH56	250:250	46.22	0.000	0.000	0.027	2.963	43.46	94.03	92433*2	87306*2	94.45
Z106BRT12	250:250	43.49	0.000	0.000	0.114	2.513	41.29	94.93	86988*2	82964*2	95.37
Z106BRT34	250:250	40.80	0.000	0.000	0.115	2.605	38.66	94.76	81602*2	77689*2	95.20
Z106BRT56	250:250	43.99	0.000	0.000	0.144	2.825	41.46	94.25	87975*2	83398*2	94.80
Z106BSO12	250:250	67.79	0.000	0.000	0.016	3.086	63.59	93.81	135575*2	127825*2	94.28
Z106BSO34	250:250	73.70	0.000	0.000	0.027	2.997	69.21	93.92	147391*2	139125*2	94.39
Z106BSO56	250:250	65.56	0.000	0.000	0.021	3.030	61.61	93.97	131117*2	123776*2	94.40
Z106CRH12	250:250	81.97	0.000	0.000	0.022	2.403	77.99	95.15	163931*2	156604*2	95.53
Z106CRH34	250:250	73.08	0.000	0.000	0.026	2.905	68.79	94.12	146168*2	138226*2	94.57
Z106CRH56	250:250	67.85	0.000	0.000	0.021	2.755	64.08	94.46	135692*2	128747*2	94.88
Z106CRT12r	250:250	29.56	0.018	0.000	0.255	1.739	28.35	95.91	59112*2	56968*2	96.37
Z106CRT34	250:250	48.93	0.000	0.000	0.096	2.287	46.64	95.31	97866*2	93725*2	95.77
Z106CRT56	250:250	46.15	0.000	0.000	0.098	2.188	44.09	95.53	92304*2	88567*2	95.95
Z106CSO12	250:250	73.37	0.000	0.000	0.023	2.614	69.48	94.70	146745*2	139602*2	95.13
Z106CSO34	250:250	74.39	0.000	0.000	0.025	2.653	70.43	94.67	148780*2	141433*2	95.06
Z106CSO56	250:250	76.85	0.000	0.000	0.025	2.813	72.52	94.36	153705*2	145679*2	94.78
Z106DRH12	250:250	65.08	0.000	0.000	0.014	2.595	61.69	94.79	130154*2	123890*2	95.19
Z106DRH34	250:250	53.33	0.000	0.000	0.022	2.630	50.47	94.63	106660*2	101451*2	95.12
Z106DRH56	250:250	66.79	0.000	0.000	0.017	2.618	63.27	94.73	133588*2	127116*2	95.16
Z106DRT12r	250:250	25.75	0.026	0.000	0.184	1.739	24.75	96.11	51494*2	49695*2	96.51
Z106DRT34	250:250	26.13	0.040	0.000	0.208	1.476	25.23	96.55	52264*2	50627*2	96.87
Z106DRT56	250:250	27.10	0.010	0.000	0.204	1.798	25.99	95.90	54197*2	52207*2	96.33
Z106DSO12	250:250	57.46	0.000	0.000	0.015	2.510	54.52	94.89	114919*2	109575*2	95.35
Z106DSO34	250:250	61.41	0.000	0.000	0.022	2.726	58.03	94.49	122824*2	116621*2	94.95
Z106DSO56	250:250	57.08	0.000	0.000	0.024	2.983	53.62	93.95	114152*2	107888*2	94.51
Z106JASO12	250:250	81.88	0.000	0.000	0.022	3.000	76.95	93.97	163760*2	154612*2	94.41
Z106JASO34	250:250	82.40	0.000	0.000	0.021	3.053	77.31	93.83	164802*2	155442*2	94.32
Z106JASO56	250:250	67.74	0.000	0.000	0.024	2.926	63.76	94.12	135478*2	128142*2	94.59
Z106JBRH12	250:250	50.25	0.000	0.000	0.027	2.802	47.41	94.35	100495*2	95241*2	94.77
Z106JBRH34	250:250	46.64	0.000	0.000	0.031	3.226	43.59	93.47	93272*2	87664*2	93.99
Z106JBRH56	250:250	49.67	0.000	0.000	0.026	2.898	46.75	94.14	99331*2	93952*2	94.58
Z106JBRT12	250:250	41.63	0.000	0.000	0.140	2.756	39.29	94.36	83269*2	78985*2	94.86
Z106JBRT34r	250:250	23.27	0.010	0.000	0.277	1.849	22.26	95.64	46548*2	44754*2	96.15
Z106JBRT56	250:250	37.07	0.000	0.000	0.139	2.167	35.39	95.47	74136*2	71114*2	95.92
Z106JBSO12	250:250	74.11	0.000	0.000	0.021	3.045	69.59	93.89	148229*2	139880*2	94.37
Z106JBSO34	250:250	67.28	0.000	0.000	0.020	3.058	63.16	93.88	134555*2	126915*2	94.32
Z106JBSO56	250:250	71.14	0.000	0.000	0.025	3.292	66.45	93.40	142280*2	133553*2	93.87
Z106JCRH12	250:250	69.94	0.000	0.000	0.025	2.940	65.84	94.15	139871*2	132287*2	94.58
Z106JCRH34	250:250	67.60	0.000	0.000	0.026	2.636	63.97	94.63	135205*2	128593*2	95.11
Z106JCRH56	250:250	70.79	0.000	0.000	0.023	2.636	67.01	94.66	141587*2	134629*2	95.09
Z106JCRT12r	250:250	26.20	0.026	0.000	0.148	1.384	25.37	96.83	52408*2	50930*2	97.18
Z106JCRT34	250:250	50.38	0.000	0.000	0.075	2.093	48.25	95.78	100757*2	96910*2	96.18
Z106JCRT56	250:250	45.70	0.000	0.000	0.106	2.563	43.32	94.78	91403*2	87106*2	95.30
Z106JCSO12	250:250	67.19	0.000	0.000	0.021	2.626	63.64	94.71	134382*2	127794*2	95.10
Z106JCSO34	250:250	73.89	0.000	0.000	0.023	2.976	69.51	94.08	147770*2	139631*2	94.49
Z106JCSO56	250:250	65.87	0.000	0.000	0.019	2.633	62.36	94.67	131734*2	125331*2	95.14
Z106JDRH12	250:250	60.19	0.000	0.000	0.020	2.345	57.33	95.25	120383*2	115171*2	95.67
Z106JDRH34	250:250	40.17	0.000	0.000	0.024	2.503	38.13	94.93	80335*2	76619*2	95.37
Z106JDRH56	250:250	43.14	0.000	0.000	0.021	2.483	40.98	95.00	86271*2	82295*2	95.39
Z106JDRT12r	250:250	28.22	0.019	0.000	0.182	1.659	27.15	96.20	56435*2	54517*2	96.60
Z106JDRT34	250:250	38.20	0.000	0.000	0.100	2.021	36.60	95.80	76401*2	73482*2	96.18
Z106JDRT56	250:250	38.13	0.000	0.000	0.103	2.084	36.49	95.69	76262*2	73335*2	96.16
Z106JDSO12	250:250	56.57	0.000	0.000	0.014	2.662	53.50	94.58	113141*2	107577*2	95.08
Z106JDSO34	250:250	67.18	0.000	0.000	0.017	2.640	63.60	94.68	134354*2	127766*2	95.10
Z106JDSO56	250:250	56.15	0.000	0.000	0.018	2.550	53.26	94.85	112301*2	106989*2	95.27

ZH10ASO12	250:250	72.42	0.000	0.000	0.022	3.419	67.46	93.15	144833*2	135735*2	93.72
ZH10ASO34	250:250	66.56	0.000	0.000	0.015	3.572	61.83	92.91	133111*2	124380*2	93.44
ZH10ASO56	250:250	69.48	0.000	0.000	0.023	3.580	64.43	92.73	138961*2	129780*2	93.39
ZH10BRH12	250:250	57.50	0.000	0.000	0.021	2.813	54.24	94.33	115003*2	109029*2	94.81
ZH10BRH34	250:250	47.88	0.000	0.000	0.026	2.992	44.99	93.96	95762*2	90416*2	94.42
ZH10BRH56	250:250	54.87	0.000	0.000	0.022	2.663	51.89	94.57	109738*2	104285*2	95.03
ZH10BRT12	250:250	42.78	0.000	0.000	0.116	2.510	40.58	94.85	85569*2	81576*2	95.33
ZH10BRT34	250:250	43.66	0.000	0.000	0.125	2.431	41.49	95.02	87325*2	83351*2	95.45
ZH10BRT56	250:250	37.60	0.000	0.000	0.153	2.699	35.48	94.35	75203*2	71347*2	94.87
ZH10BSO12	250:250	74.50	0.000	0.000	0.025	3.381	69.48	93.27	148997*2	139664*2	93.74
ZH10BSO34	250:250	63.98	0.000	0.000	0.037	3.622	59.31	92.70	127966*2	119327*2	93.25
ZH10BSO56	250:250	74.49	0.000	0.000	0.017	3.204	69.72	93.59	148980*2	140183*2	94.10
ZH10CRH12	250:250	71.57	0.000	0.000	0.023	2.584	67.85	94.80	143145*2	136286*2	95.21
ZH10CRH34	250:250	74.91	0.001	0.000	0.027	2.903	70.53	94.16	149814*2	141744*2	94.61
ZH10CRH56	250:250	75.14	0.000	0.000	0.020	2.720	70.99	94.48	150271*2	142660*2	94.94
ZH10CRT12	250:250	47.03	0.000	0.000	0.085	2.047	45.05	95.78	94064*2	90474*2	96.18
ZH10CRT34	250:250	36.14	0.000	0.000	0.153	2.447	34.28	94.87	72278*2	68903*2	95.33
ZH10CRT56	250:250	48.89	0.000	0.000	0.085	2.046	46.84	95.79	97783*2	94053*2	96.19
ZH10CSO12	250:250	65.56	0.000	0.000	0.023	2.722	61.92	94.44	131121*2	124453*2	94.91
ZH10CSO34	250:250	74.71	0.000	0.000	0.018	2.746	70.57	94.47	149411*2	141815*2	94.92
ZH10CSO56	250:250	69.91	0.000	0.000	0.021	2.632	66.20	94.69	139828*2	133000*2	95.12
ZH10DRH12	250:250	76.00	0.000	0.000	0.017	2.466	72.21	95.01	152003*2	145012*2	95.40
ZH10DRH34	250:250	60.05	0.000	0.000	0.020	2.400	57.11	95.10	120106*2	114722*2	95.52
ZH10DRH56	250:250	67.27	0.000	0.000	0.023	2.423	63.96	95.08	134531*2	128443*2	95.47
ZH10DRT12r	250:250	25.68	0.007	0.000	0.164	1.567	24.77	96.45	51356*2	49733*2	96.84
ZH10DRT34	250:250	44.28	0.000	0.000	0.092	1.912	42.50	95.99	88555*2	85364*2	96.40
ZH10DRT56	250:250	41.15	0.000	0.000	0.101	1.823	39.57	96.16	82298*2	79439*2	96.53
ZH10DSO12	250:250	49.78	0.000	0.000	0.016	2.742	47.01	94.44	99551*2	94513*2	94.94
ZH10DSO34	250:250	49.51	0.000	0.000	0.017	2.656	46.85	94.61	99024*2	94145*2	95.07
ZH10DSO56	250:250	44.88	0.000	0.000	0.014	2.766	42.37	94.41	89756*2	85171*2	94.89
Z106CRT12r*	250:250	30.67	0.013	0.000	0.084	2.422	28.98	94.49	61344*2	58686*2	95.67
Z106DRT12r*	250:250	27.73	0.017	0.000	0.079	2.179	26.34	95.01	55453*2	53260*2	96.05
Z106DRT34*	250:250	34.50	0.010	0.000	0.077	1.900	33.00	95.66	69000*2	66553*2	96.45
Z106DRT56*	250:250	29.46	0.013	0.000	0.062	2.223	27.95	94.88	58924*2	56548*2	95.97
Z106JBRT34r*	250:250	25.66	0.008	0.000	0.073	2.868	24.03	93.63	51329*2	48698*2	94.87
Z106JBRT56*	250:250	37.36	0.003	0.000	0.103	2.683	35.08	93.90	74713*2	71101*2	95.17
Z106JCRT12r*	250:250	27.52	0.007	0.000	0.053	1.983	26.29	95.53	55030*2	53091*2	96.48
Z106JDRT12r*	250:250	28.89	0.012	0.000	0.056	2.273	27.41	94.89	57782*2	55423*2	95.92
Z106JDRT34*	250:250	43.53	0.002	0.000	0.069	1.990	41.58	95.53	87062*2	83890*2	96.36
Z106JDRT56*	250:250	45.03	0.005	0.000	0.071	2.125	42.83	95.09	90070*2	86605*2	96.15
ZH10BRT56*	250:250	41.65	0.004	0.000	0.096	3.471	38.41	92.22	83302*2	78120*2	93.78
ZH10CRT34*	250:250	33.51	0.004	0.000	0.107	3.001	31.22	93.16	67017*2	63331*2	94.50
ZH10DRT12r*	250:250	28.06	0.009	0.000	0.053	2.362	26.59	94.79	56111*2	53768*2	95.82

The 13 samples marked with ‘*’ are the new extraction and sequencing of the corresponding samples in the first 90 samples, which were uploaded with the corresponding samples together.

Table S2: ***P*-values of six indices for alpha diversity of the different compartment samples between ZH10, Z106, and Z106J according to Student's *t*-test**

Group vs. Group	*p*-value					
	sobs	shannon	simpson	ace	chao	coverage
ZH10ASO vs Z106ASO	0.06484	0.1057	0.07645	0.06644	0.07312	0.03884
ZH10ASO vs Z106JASO	0.3245	0.3622	0.5986	0.2409	0.6143	0.5679
Z106ASO vs Z106JASO	0.09639	0.1668	0.2356	0.1505	0.07041	0.07143
ZH10BSO vs Z106BSO	0.2821	0.291	0.3074	0.1935	0.2522	0.2208
ZH10BSO vs Z106JBSO	0.1262	0.1527	0.07814	0.05668	0.07047	0.04921
Z106BSO vs Z106JBSO	0.6248	0.5122	0.04269	0.7853	0.8716	0.7841
ZH10BRH vs Z106BRH	0.6411	0.6735	0.7376	0.4808	0.8923	0.6338
ZH10BRH vs Z106JBRH	0.3026	0.376	0.6169	0.668	0.6685	0.4226
Z106BRH vs Z106JBRH	0.725	0.7547	0.9	0.8484	0.8058	0.8032
ZH10BRT vs Z106BRT	0.2782	0.6666	0.6091	0.2481	0.2972	0.1926
ZH10BRT vs Z106JBRT	0.3431	0.4095	0.3882	0.06573	0.05405	0.1213
Z106BRT vs Z106JBRT	0.8866	0.3496	0.2952	0.1347	0.08522	0.479
ZH10CSO vs Z106CSO	0.8673	0.4141	0.2619	0.9125	0.9639	0.7957
ZH10CSO vs Z106JCSO	0.9712	0.582	0.3435	0.7442	0.7277	0.8751
Z106CSO vs Z106JCSO	0.7536	0.3701	0.2018	0.6251	0.4811	0.8604
ZH10CRH vs Z106CRH	0.9194	0.5748	0.4931	0.3874	0.9239	0.6042
ZH10CRH vs Z106JCRH	0.7168	0.7078	0.8576	0.6191	0.4943	0.6303
Z106CRH vs Z106JCRH	0.7444	0.4826	0.411	0.9247	0.4787	0.8333
ZH10CRT vs Z106CRT	0.4309	0.03484	0.1348	0.8385	0.6895	0.6696
ZH10CRT vs Z106JCRT	0.6189	0.5548	0.6827	0.4378	0.5784	0.5906
Z106CRT vs Z106JCRT	0.7673	0.3304	0.3115	0.6998	0.9764	0.9961
ZH10DSO vs Z106DSO	0.9212	0.5109	0.3798	0.1926	0.05466	0.2498
ZH10DSO vs Z106JDSO	0.2275	0.3215	0.5231	0.2904	0.06129	0.2035
Z106DSO vs Z106JDSO	0.1937	0.1159	0.08963	0.492	0.7623	0.5334
ZH10DRH vs Z106DRH	0.01729	0.1108	0.287	0.3773	0.4174	0.1111
ZH10DRH vs Z106JDRH	0.6389	0.8041	0.8867	0.2382	0.676	0.4581
Z106DRH vs Z106JDRH	0.395	0.484	0.4921	0.6224	0.8996	0.7527
ZH10DRT vs Z106DRT	0.3086	0.5827	0.7451	0.6022	0.4238	0.3388
ZH10DRT vs Z106JDRT	0.7895	0.6136	0.5852	0.939	0.9707	0.9865
Z106DRT vs Z106JDRT	0.1303	0.1889	0.2975	0.6471	0.4189	0.2709

The *p*-values in red color ($p < 0.05$) indicate significant difference.

Table S3: ANOSIM and Adonis of the different compartment samples between ZH10, Z106, and Z106J based on the Bray–Curtis, Weighted Unifrac and Unweighted Unifrac distance metrics

Distance metrics	Group vs. Group	Adonis		ANOSIM	
		R^2	*p*-value	Statistic	*p*-value
Bray–Curtis	ZH10ASO vs Z106ASO	0.22082	0.1	0.2963	0.098
	ZH10ASO vs Z106JASO	0.21999	0.1	0.1111	0.287
	Z106ASO vs Z106JASO	0.22283	0.1	0.1852	0.098
	ZH10BSO vs Z106BSO	0.20927	0.3	0.2222	0.296
	ZH10BSO vs Z106JBSO	0.22953	0.2	0.2963	0.289
	Z106BSO vs Z106JBSO	0.20750	0.2	0.0556	0.195
	ZH10CSO vs Z106CSO	0.18958	0.9	-0.1111	0.908
	ZH10CSO vs Z106JCSO	0.21038	0.2	0.0741	0.301
	Z106CSO vs Z106JCSO	0.20109	0.3	-0.1481	0.787
	ZH10DSO vs Z106DSO	0.20022	0.5	0.037	0.394
	ZH10DSO vs Z106JDSO	0.19960	0.6	-0.0741	0.723
	Z106DSO vs Z106JDSO	0.20774	0.2	0.2593	0.209
	ZH10BRH vs Z106BRH	0.17251	0.9	-0.1852	0.703
	ZH10BRH vs Z106JBRH	0.17604	0.8	-0.3333	1
	Z106BRH vs Z106JBRH	0.18659	0.9	-0.0556	0.588
	ZH10CRH vs Z106CRH	0.20792	0.3	0.037	0.298
	ZH10CRH vs Z106JCRH	0.20552	0.5	-0.037	0.597
	Z106CRH vs Z106JCRH	0.19412	0.6	-0.037	0.615
	ZH10DRH vs Z106DRH	0.19691	0.4	-0.0741	0.592
	ZH10DRH vs Z106JDRH	0.21890	0.2	0.1852	0.195
	Z106DRH vs Z106JDRH	0.21390	0.4	0.2963	0.098
	ZH10BRT vs Z106BRT	0.17920	0.5	-0.037	0.519
	ZH10BRT vs Z106JBRT	0.14906	1	-0.1111	0.809
	Z106BRT vs Z106JBRT	0.22438	0.4	0.1111	0.403
	ZH10CRT vs Z106CRT	0.27254	0.3	0.2222	0.287
	ZH10CRT vs Z106JCRT	0.10615	0.9	-0.4074	1
	Z106CRT vs Z106JCRT	0.15171	0.7	-0.2593	0.908
	ZH10DRT vs Z106DRT	0.16544	0.7	-0.1852	0.691
	ZH10DRT vs Z106JDRT	0.13188	0.7	-0.1111	0.699
	Z106DRT vs Z106JDRT	0.20956	0.5	0	0.594

Distance metrics	Group vs. Group	Adonis		ANOSIM	
		R^2	*p*-value	Statistic	*p*-value
Weighted Unifrac	ZH10ASO vs Z106ASO	0.25767	0.2	0.1852	0.195
	ZH10ASO vs Z106JASO	0.26509	0.1	0.2593	0.195
	Z106ASO vs Z106JASO	0.24187	0.2	0.2593	0.098
	ZH10BSO vs Z106BSO	0.20198	0.5	-0.037	0.496
	ZH10BSO vs Z106JBSO	0.27467	0.2	0.2593	0.289
	Z106BSO vs Z106JBSO	0.18180	0.4	-0.2222	0.811
	ZH10CSO vs Z106CSO	0.18068	0.8	0.0741	0.298
	ZH10CSO vs Z106JCSO	0.18007	0.6	0.0741	0.404
	Z106CSO vs Z106JCSO	0.20562	0.4	0.0741	0.384
	ZH10DSO vs Z106DSO	0.20540	0.4	-0.037	0.582
	ZH10DSO vs Z106JDSO	0.23807	0.3	0.2222	0.305
	Z106DSO vs Z106JDSO	0.32242	0.2	0.5556	0.209
	ZH10BRH vs Z106BRH	0.13747	0.8	-0.2222	0.8
	ZH10BRH vs Z106JBRH	0.13365	0.8	-0.2593	0.899
	Z106BRH vs Z106JBRH	0.10437	0.9	-0.1852	0.809
	ZH10CRH vs Z106CRH	0.18424	0.6	-0.037	0.59
	ZH10CRH vs Z106JCRH	0.19792	0.5	0	0.494
	Z106CRH vs Z106JCRH	0.11793	1	-0.2963	1
	ZH10DRH vs Z106DRH	0.17162	0.6	-0.2593	1
	ZH10DRH vs Z106JDRH	0.23970	0.3	0.3333	0.201
	Z106DRH vs Z106JDRH	0.27017	0.2	0.1481	0.195
	ZH10BRT vs Z106BRT	0.18073	0.5	0.2222	0.209
	ZH10BRT vs Z106JBRT	0.14246	0.8	0	0.708
	Z106BRT vs Z106JBRT	0.42320	0.1	0.5926	0.098
	ZH10CRT vs Z106CRT	0.29837	0.2	0.1481	0.498
	ZH10CRT vs Z106JCRT	0.07181	1	-0.3333	1
	Z106CRT vs Z106JCRT	0.13213	0.7	-0.1481	0.71
	ZH10DRT vs Z106DRT	0.17316	0.6	-0.0741	0.581
	ZH10DRT vs Z106JDRT	0.08363	1	-0.2593	1
	Z106DRT vs Z106JDRT	0.18573	0.6	0.037	0.594

Distance metrics	Group vs. Group	Adonis		ANOSIM	
		R^2	*P*-value	Statistic	*P*-value
Unweighted Unifrac	ZH10ASO vs Z106ASO	0.20000	0.6	0	0.612
	ZH10ASO vs Z106JASO	0.20081	0.6	0.1481	0.413
	Z106ASO vs Z106JASO	0.19285	1	-0.1481	0.912
	ZH10BSO vs Z106BSO	0.20117	0.6	0.1481	0.499
	ZH10BSO vs Z106JBSO	0.19366	0.9	-0.3333	0.889
	Z106BSO vs Z106JBSO	0.20312	0.3	0.1111	0.602
	ZH10CSO vs Z106CSO	0.20012	0.5	-0.037	0.714
	ZH10CSO vs Z106JCSO	0.20202	0.4	0.1111	0.386
	Z106CSO vs Z106JCSO	0.21422	0.1	0.4444	0.098
	ZH10DSO vs Z106DSO	0.20438	0.3	-0.037	0.714
	ZH10DSO vs Z106JDSO	0.20637	0.2	0.1852	0.304
	Z106DSO vs Z106JDSO	0.21537	0.1	0.6667	0.098
	ZH10BRH vs Z106BRH	0.20152	0.5	-0.037	0.786
	ZH10BRH vs Z106JBRH	0.19472	0.8	-0.1481	0.699
	Z106BRH vs Z106JBRH	0.19482	0.8	-0.2222	0.897
	ZH10CRH vs Z106CRH	0.20639	0.2	0.1852	0.201
	ZH10CRH vs Z106JCRH	0.19021	0.9	-0.3333	1
	Z106CRH vs Z106JCRH	0.18843	1	-0.2222	0.903
	ZH10DRH vs Z106DRH	0.20262	0.4	0.037	0.319
	ZH10DRH vs Z106JDRH	0.21310	0.1	0.2593	0.201
	Z106DRH vs Z106JDRH	0.21014	0.2	0.1111	0.384
	ZH10BRT vs Z106BRT	0.19901	0.7	0.037	0.306
	ZH10BRT vs Z106JBRT	0.15775	1	-0.3333	1
	Z106BRT vs Z106JBRT	0.19334	0.8	0	0.62
	ZH10CRT vs Z106CRT	0.16473	0.7	-0.1481	0.595
	ZH10CRT vs Z106JCRT	0.17699	0.7	-0.1852	0.698
	Z106CRT vs Z106JCRT	0.16755	0.5	-0.1852	0.712
	ZH10DRT vs Z106DRT	0.18471	1	-0.2222	1
	ZH10DRT vs Z106JDRT	0.18383	0.9	-0.2593	0.889
	Z106DRT vs Z106JDRT	0.19786	0.6	0.037	0.482

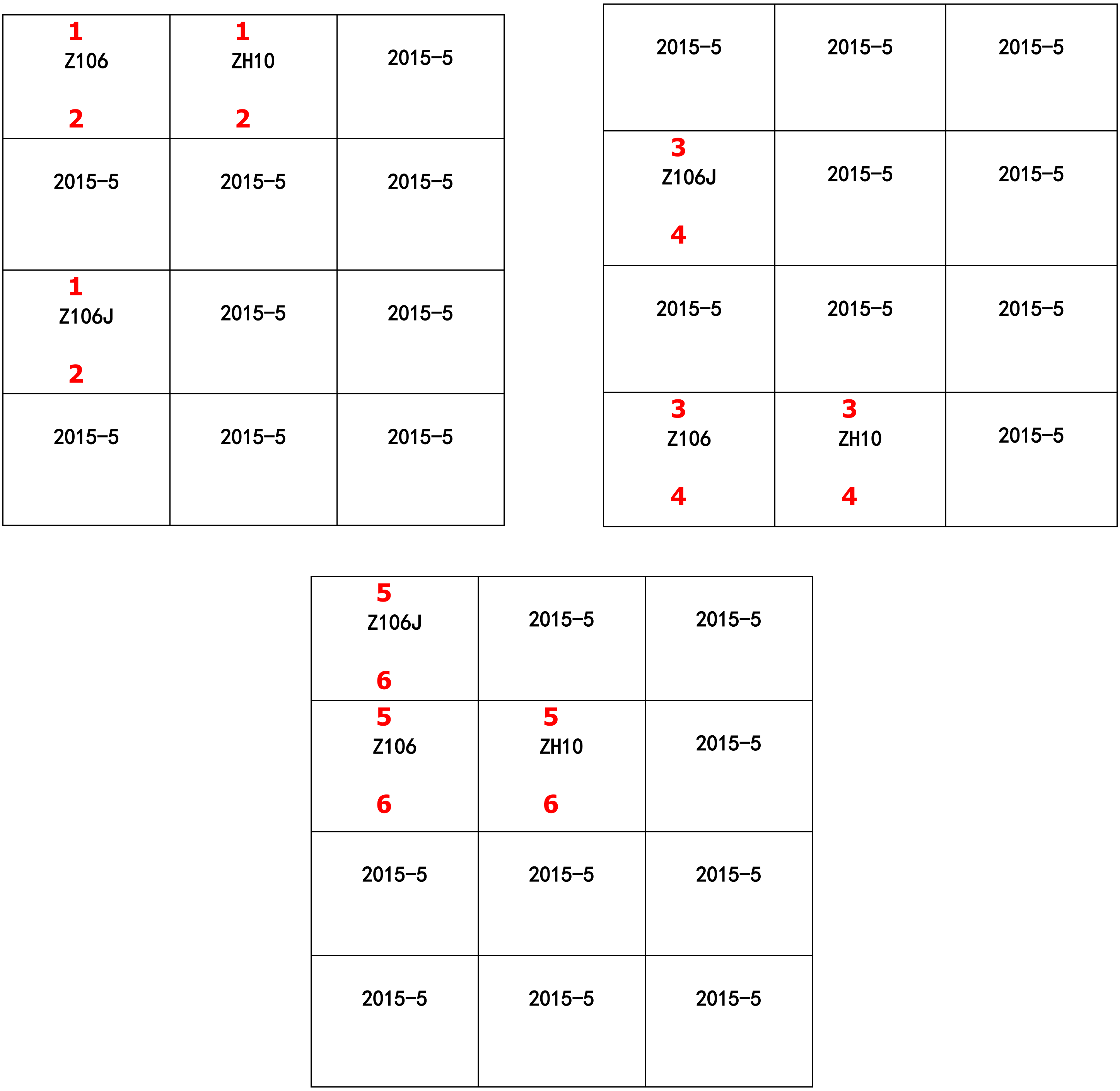

Fig. S1: Field design and sampling sites. Z106 and ZH10 represent the transgenic soybean (ZH10-6) and its recipient cultivar (Zhonghuang 10), respectively. Z106J represents the Z106 treated with glyphosate application. 2015-5 represents the local soybean cultivar which not used in our study. The red numbers represent sampling sites, and composite samples from 2 sampling sites per plot were made and treated as one replicate.

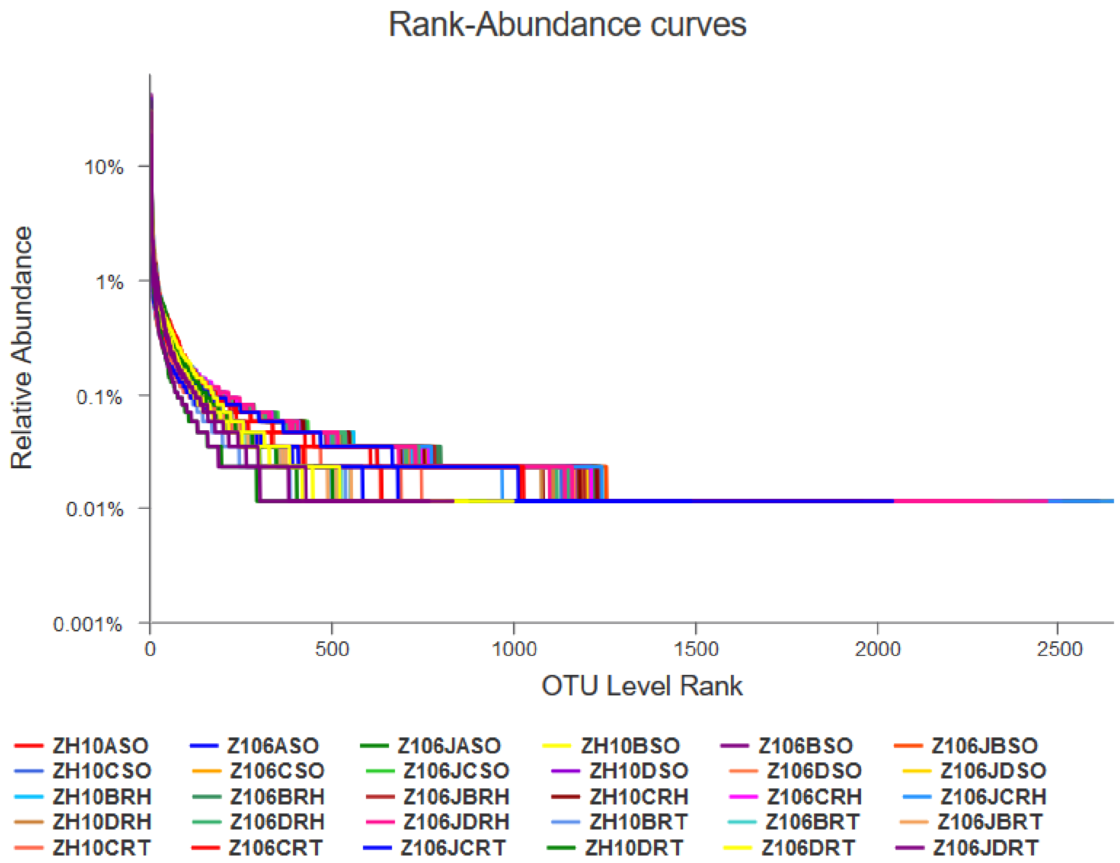

Fig. S2: Rank-Abundance curves of all 90 samples.

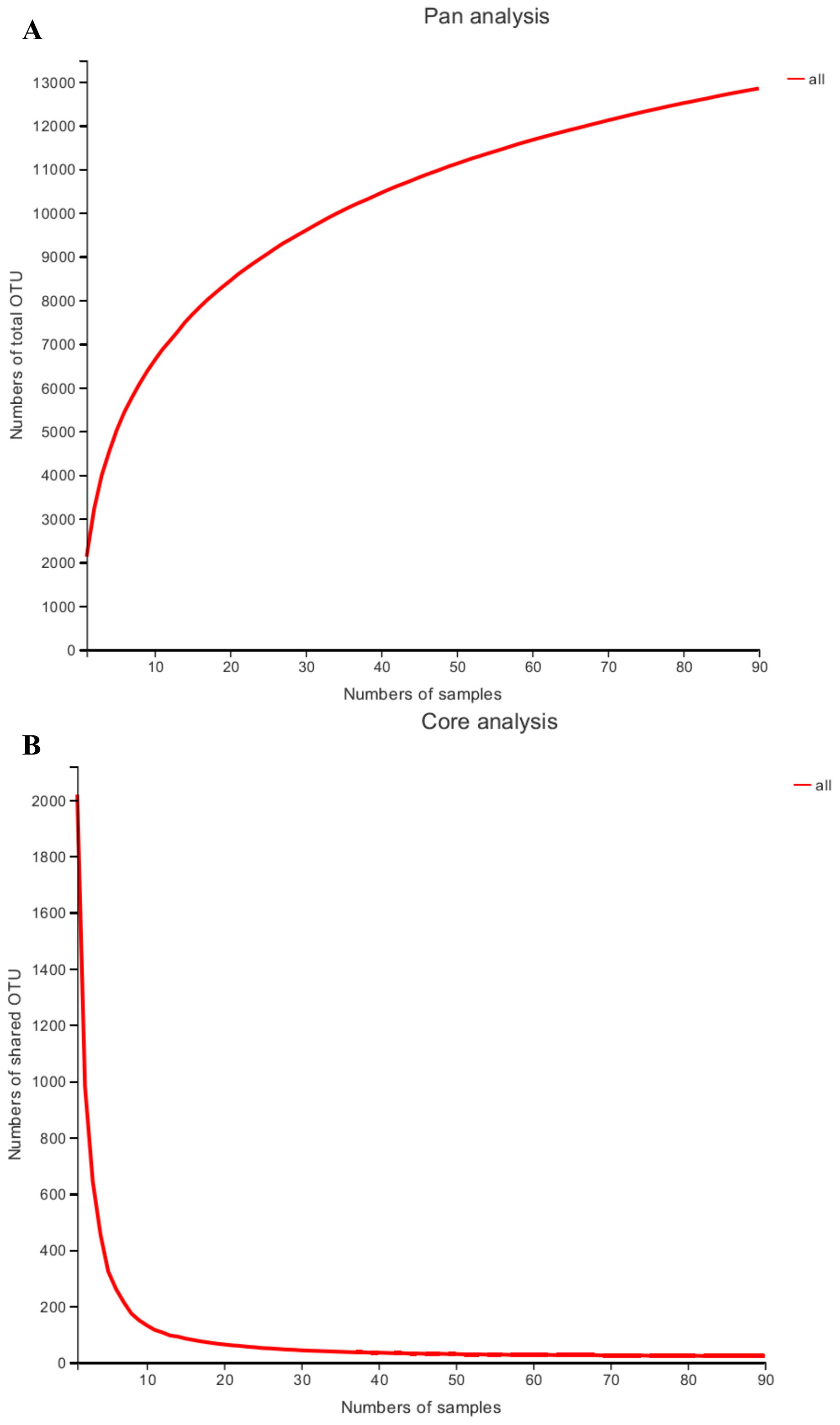

Fig. S3: Pan-Core analysis of all samples. A indicates Pan analysis, B indicates Core analysis. The total number of species in all samples is 12847 and the number of core species is 23.

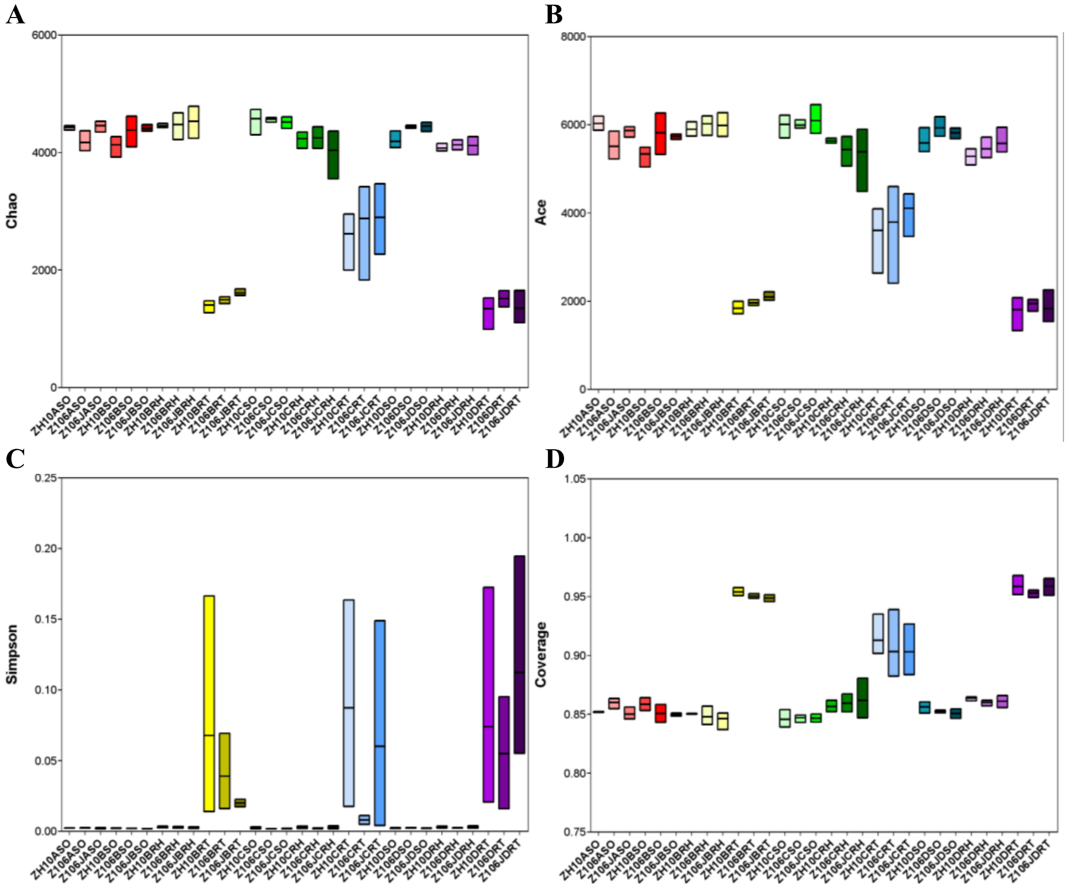

Fig. S4: The remaining boxplot of alpha diversity of Chao index (A), Ace index (B), Simpson index (C), and Good's coverage (D).

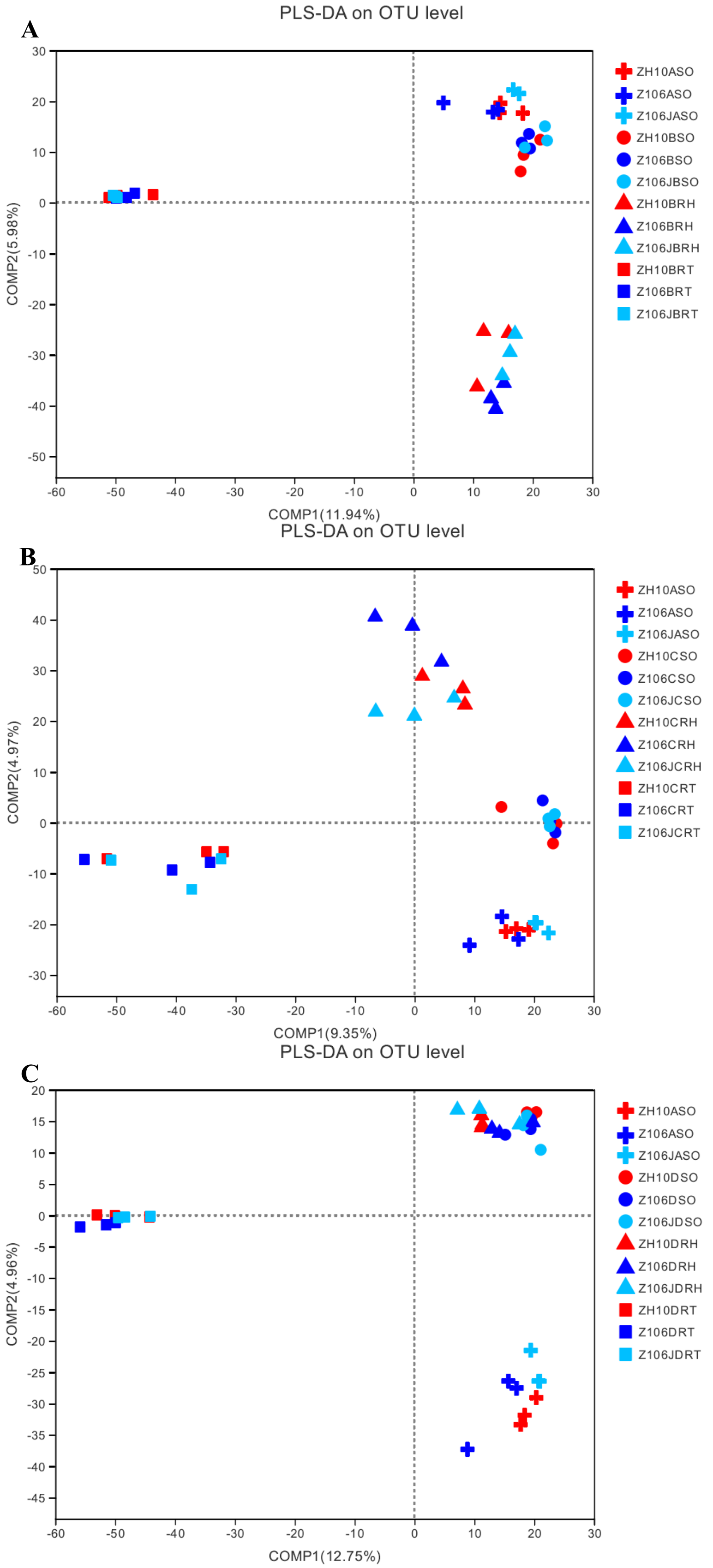

Fig. S5: Partial Least Squares Discriminant Analysis on OTU level at seedling (A), flowering (B) and seed-filling (C) stages.

A

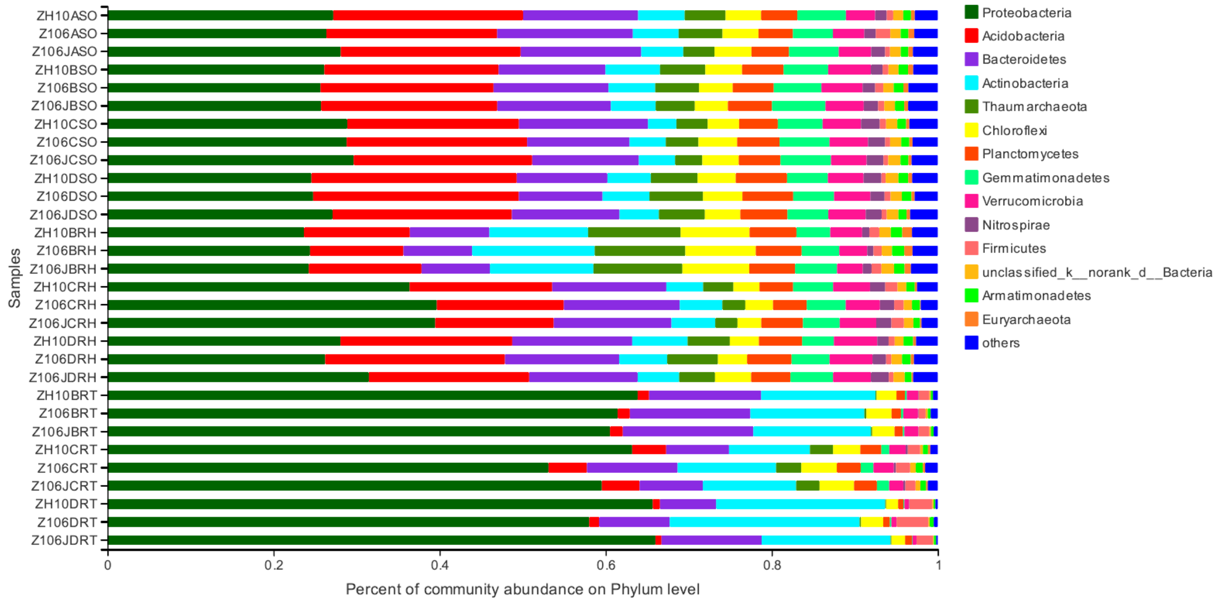
ZH10ASO
Z106ASO
Z106JASO
ZH10BSO
Z106BSO
Z106JBSO
ZH10CSO
Z106CSO
Z106JCSO
ZH10DSO
Z106DSO
Z106JDSO
ZH10BRH
Z106BRH
Z106JBRH
ZH10CRH
Z106CRH
Z106JCRH
ZH10DRH
Z106DRH
Z106JDRH
ZH10BRT
Z106BRT
Z106JBRT
ZH10CRT
Z106CRT
Z106JCRT
ZH10DRT
Z106DRT
Z106JDRT
Samples
0
0.2
0.4
0.6
0.8
1
Percent of community abundance on Phylum level
Proteobacteria
Acidobacteria
Bacteroidetes
Actinobacteria
Thaumarchaeota
Chloroflexi
Planctomycetes
Gemmatimonadetes
Verrucomicrobia
Nitrospirae
Firmicutes
unclassified_k__norank_d__Bacteria
Armatimonadetes
Euryarchaeota
others

B

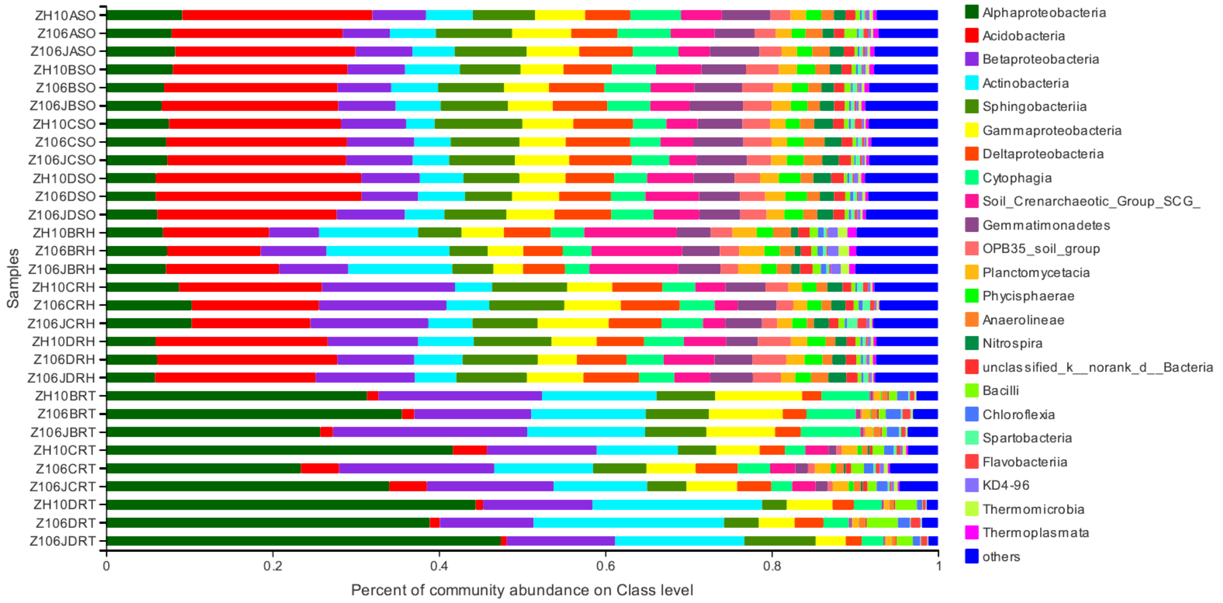
ZH10ASO
Z106ASO
Z106JASO
ZH10BSO
Z106BSO
Z106JBSO
ZH10CSO
Z106CSO
Z106JCSO
ZH10DSO
Z106DSO
Z106JDSO
ZH10BRH
Z106BRH
Z106JBRH
ZH10CRH
Z106CRH
Z106JCRH
ZH10DRH
Z106DRH
Z106JDRH
ZH10BRT
Z106BRT
Z106JBRT
ZH10CRT
Z106CRT
Z106JCRT
ZH10DRT
Z106DRT
Z106JDRT
Samples
0
0.2
0.4
0.6
0.8
1
Percent of community abundance on Class level
Alphaproteobacteria
Acidobacteria
Betaproteobacteria
Actinobacteria
Sphingobacteriia
Gammaproteobacteria
Deltaproteobacteria
Cytophagia
Soil_Crenarchaeotic_Group_SCG_
Gemmatimonadetes
OPB35_soil_group
Planctomycetacia
Phycisphaerae
Anaerolineae
Nitrospira
unclassified_k__norank_d__Bacteria
Bacilli
Chloroflexia
Spartobacteria
Flavobacteriia
KD4-96
Thermomicrobia
Thermoplasmata
others

C

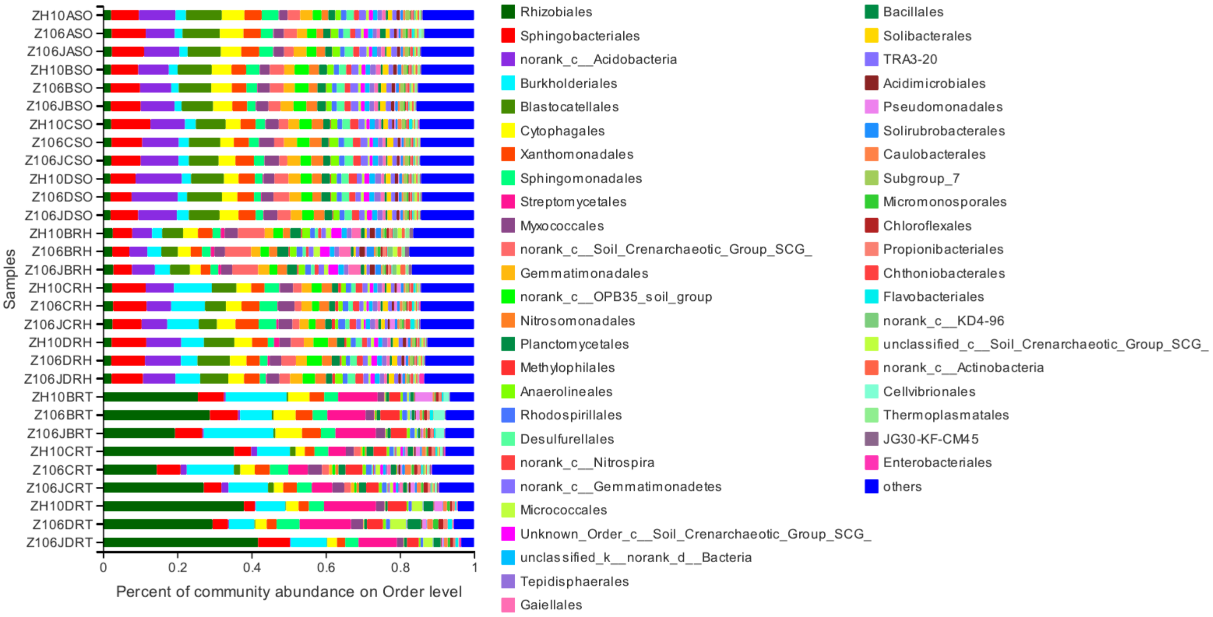
ZH10ASO
Z106ASO
Z106JASO
ZH10BSO
Z106BSO
Z106JBSO
ZH10CSO
Z106CSO
Z106JCSO
ZH10DSO
Z106DSO
Z106JDSO
ZH10BRH
Z106BRH
Z106JBRH
ZH10CRH
Z106CRH
Z106JCRH
ZH10DRH
Z106DRH
Z106JDRH
ZH10BRT
Z106BRT
Z106JBRT
ZH10CRT
Z106CRT
Z106JCRT
ZH10DRT
Z106DRT
Z106JDRT
Samples
0
0.2
0.4
0.6
0.8
1
Percent of community abundance on Order level
Rhizobiales
Sphingobacteriales
norank_c__Acidobacteria
Burkholderiales
Blastocatellales
Cytophagales
Xanthomonadales
Sphingomonadales
Streptomycetales
Myxococcales
norank_c__Soil_Crenarchaeotic_Group_SCG_
Gemmatimonadales
norank_c__OPB35_soil_group
Nitrosomonadales
Planctomycetales
Methylophilales
Anaerolineales
Rhodospirillales
Desulfurellales
norank_c__Nitrospira
norank_c__Gemmatimonadetes
Micrococcales
Unknown_Order_c__Soil_Crenarchaeotic_Group_SCG_
unclassified_k__norank_d__Bacteria
Tepidisphaerales
Gaiellales
Bacillales
Solibacterales
TRA3-20
Acidimicrobiales
Pseudomonadales
Solirubrobacterales
Caulobacterales
Subgroup_7
Micromonosporales
Chloroflexales
Propionibacteriales
Chthoniobacterales
Flavobacteriales
norank_c__KD4-96
unclassified_c__Soil_Crenarchaeotic_Group_SCG_
norank_c__Actinobacteria
Cellvibrionales
Thermoplasmatales
JG30-KF-CM45
Enterobacteriales
others

D

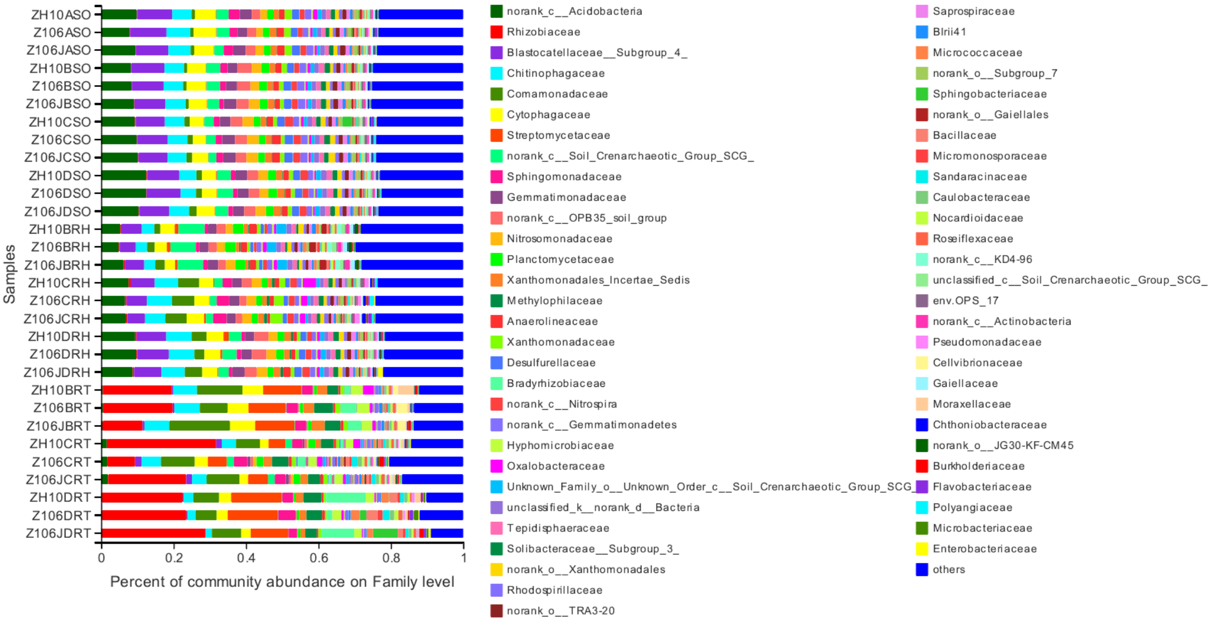
ZH10ASO
Z106ASO
Z106JASO
ZH10BSO
Z106BSO
Z106JBSO
ZH10CSO
Z106CSO
Z106JCSO
ZH10DSO
Z106DSO
Z106JDSO
ZH10BRH
Z106BRH
Z106JBRH
ZH10CRH
Z106CRH
Z106JCRH
ZH10DRH
Z106DRH
Z106JDRH
ZH10BRT
Z106BRT
Z106JBRT
ZH10CRT
Z106CRT
Z106JCRT
ZH10DRT
Z106DRT
Z106JDRT
Samples
0
0.2
0.4
0.6
0.8
1
Percent of community abundance on Family level
norank_c__Acidobacteria
Rhizobiaceae
Blastocatellaceae__Subgroup_4_
Chitinophagaceae
Comamonadaceae
Cytophagaceae
Streptomycetaceae
norank_c__Soil_Crenarchaeotic_Group_SCG_
Sphingomonadaceae
Gemmatimonadaceae
norank_c__OPB35_soil_group
Nitrosomonadaceae
Planctomycetaceae
Xanthomonadales_Incertae_Sedis
Methylophilaceae
Anaerolineaceae
Xanthomonadaceae
Desulfurellaceae
Bradyrhizobiaceae
norank_c__Nitrospira
norank_c__Gemmatimonadetes
Hyphomicrobiaceae
Oxalobacteraceae
Unknown_Family_o__Unknown_Order_c__Soil_Crenarchaeotic_Group_SCG_
unclassified_k__norank_d__Bacteria
Tepidisphaeraceae
Solibacteraceae__Subgroup_3_
norank_o__Xanthomonadales
Rhodospirillaceae
norank_o__TRA3-20
Saprospiraceae
Blrii41
Micrococcaceae
norank_o__Subgroup_7
Sphingobacteriaceae
norank_o__Gaiellales
Bacillaceae
Micromonosporaceae
Sandaracinaceae
Caulobacteraceae
Nocardioidaceae
Roseiflexaceae
norank_c__KD4-96
unclassified_c__Soil_Crenarchaeotic_Group_SCG_
env.OPS_17
norank_c__Actinobacteria
Pseudomonadaceae
Cellvibrionaceae
Gaiellaceae
Moraxellaceae
Chthoniobacteraceae
norank_o__JG30-KF-CM45
Burkholderiaceae
Flavobacteriaceae
Polyangiaceae
Microbacteriaceae
Enterobacteriaceae
others

E

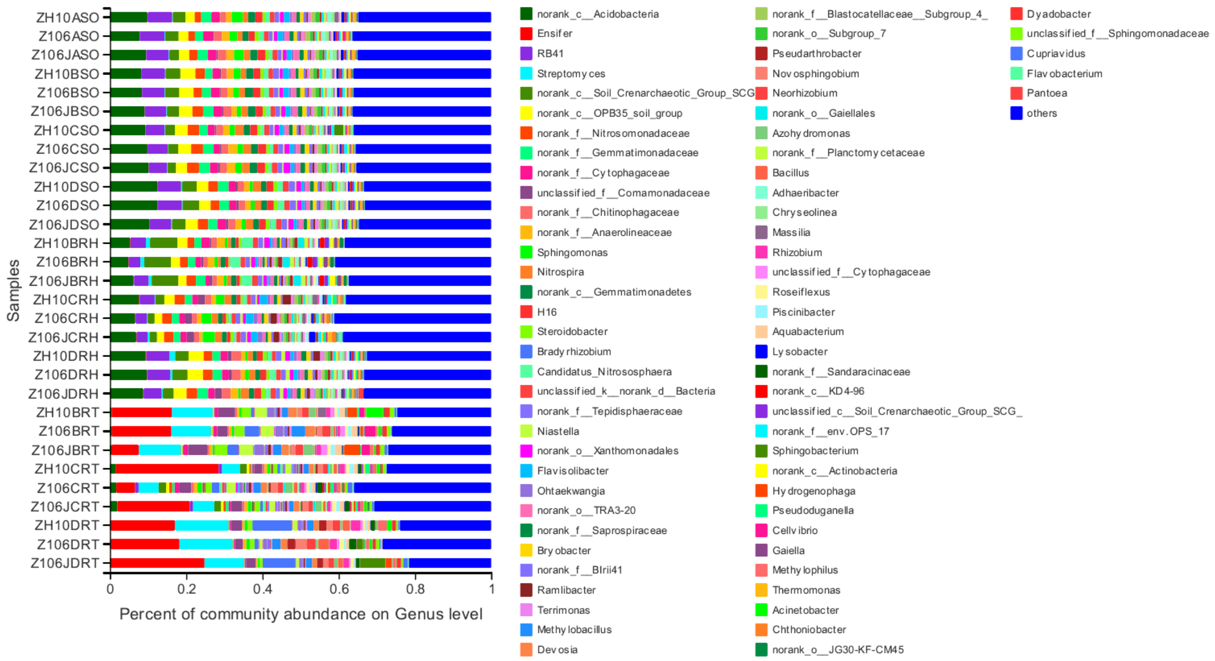

F

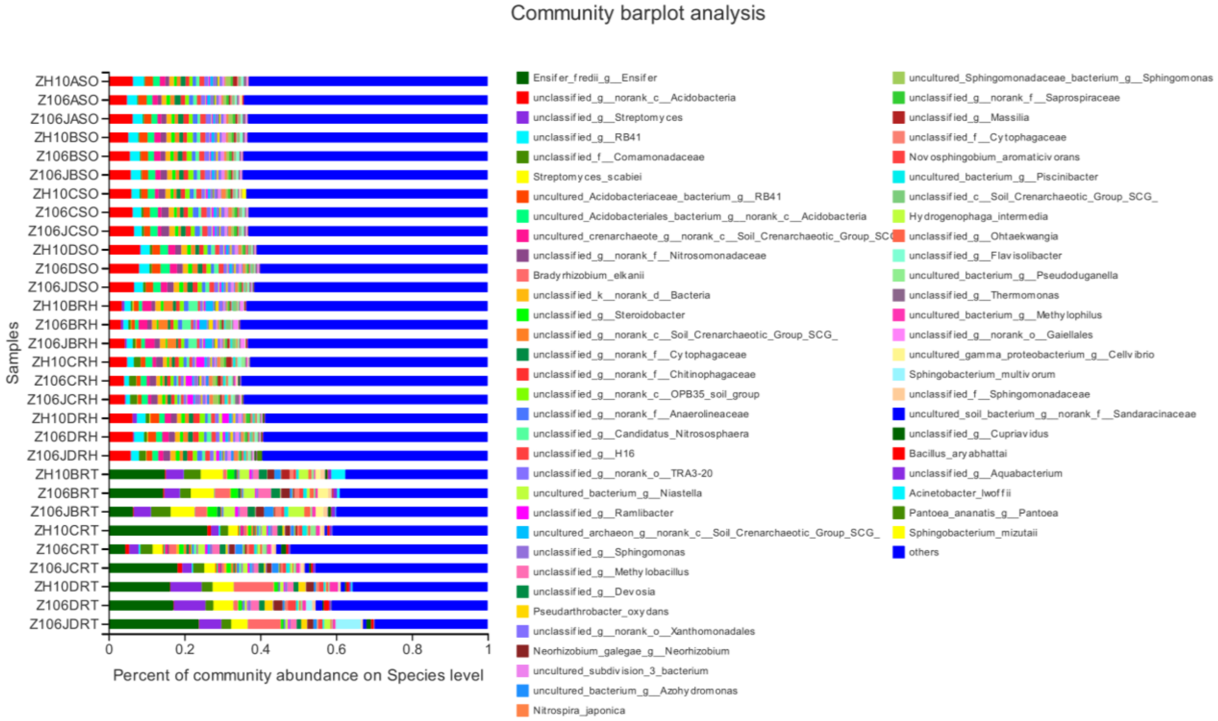

Fig. S6: Community barplot analysis of all samples. A, B, C, D, E, F indicate Phylum, Class, Order, Family, Genus and Species.

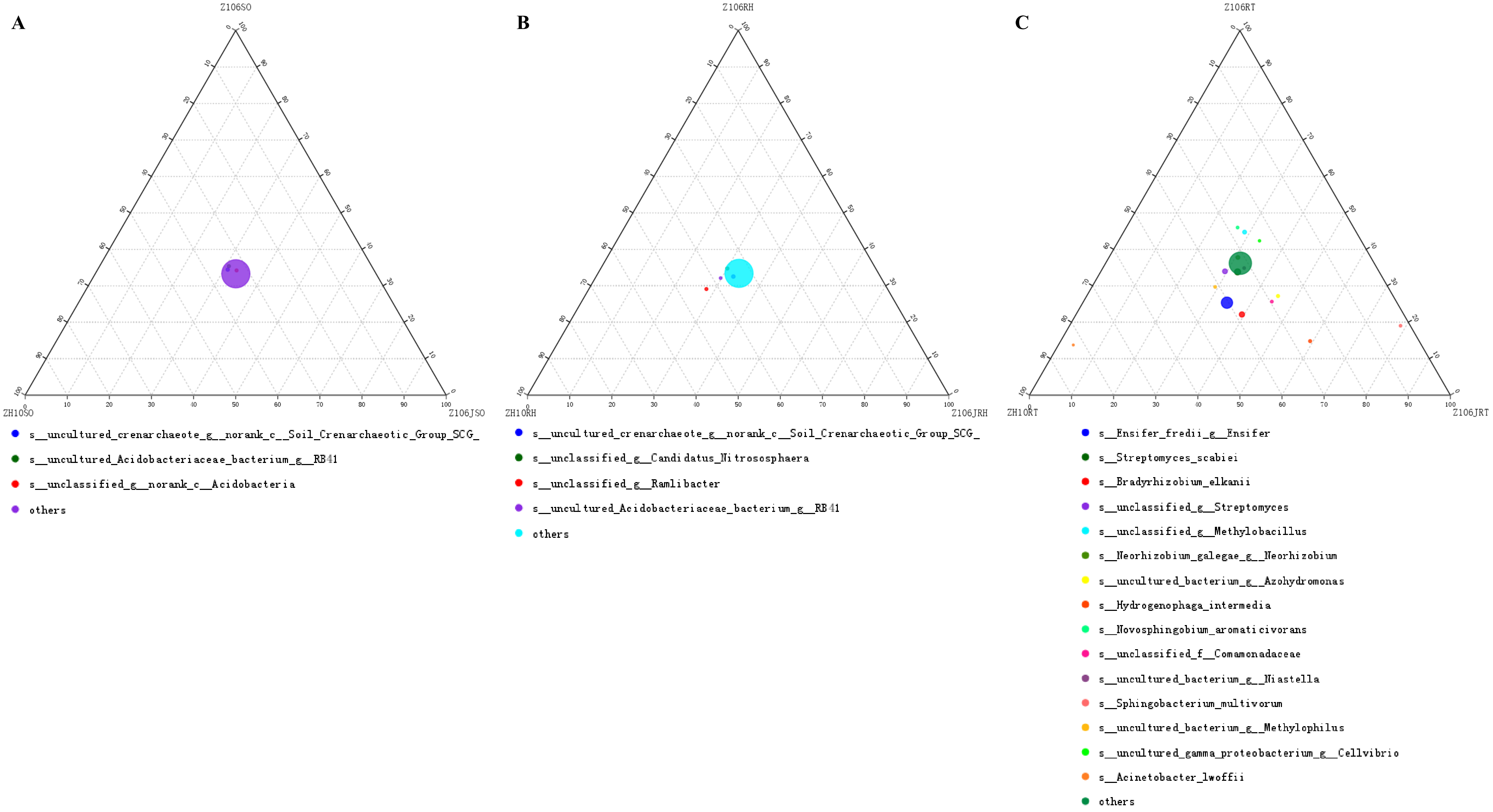
A
Z106SO
ZH10SO
Z106JSO
s__uncultured_crenarchaeote_g__norank_c__Soil_Crenarchaeotic_Group_SCG_
s__uncultured_Acidobacteriaceae_bacterium_g__RB41
s__unclassified_g__norank_c__Acidobacteria
others
B
Z106RH
ZH10RH
Z106JRH
s__uncultured_crenarchaeote_g__norank_c__Soil_Crenarchaeotic_Group_SCG_
s__unclassified_g__Candidatus_Nitrososphaera
s__unclassified_g__Ramlibacter
s__uncultured_Acidobacteriaceae_bacterium_g__RB41
others
C
Z106RT
ZH10RT
Z106JRT
s__Ensifer_fredii_g__Ensifer
s__Streptomyces_scabiei
s__Bradyrhizobium_elkanii
s__unclassified_g__Streptomyces
s__unclassified_g__Methylobacillus
s__Neorhizobium_galegae_g__Neorhizobium
s__uncultured_bacterium_g__Azohydromonas
s__Hydrogenophaga_intermedia
s__Novosphingobium_aromaticivorans
s__unclassified_f__Comamonadaceae
s__uncultured_bacterium_g__Niastella
s__Sphingobacterium_multivorum
s__uncultured_bacterium_g__Methylophilus
s__uncultured_gamma_proteobacterium_g__Cellvibrio
s__Acinetobacter_lwoffii
others

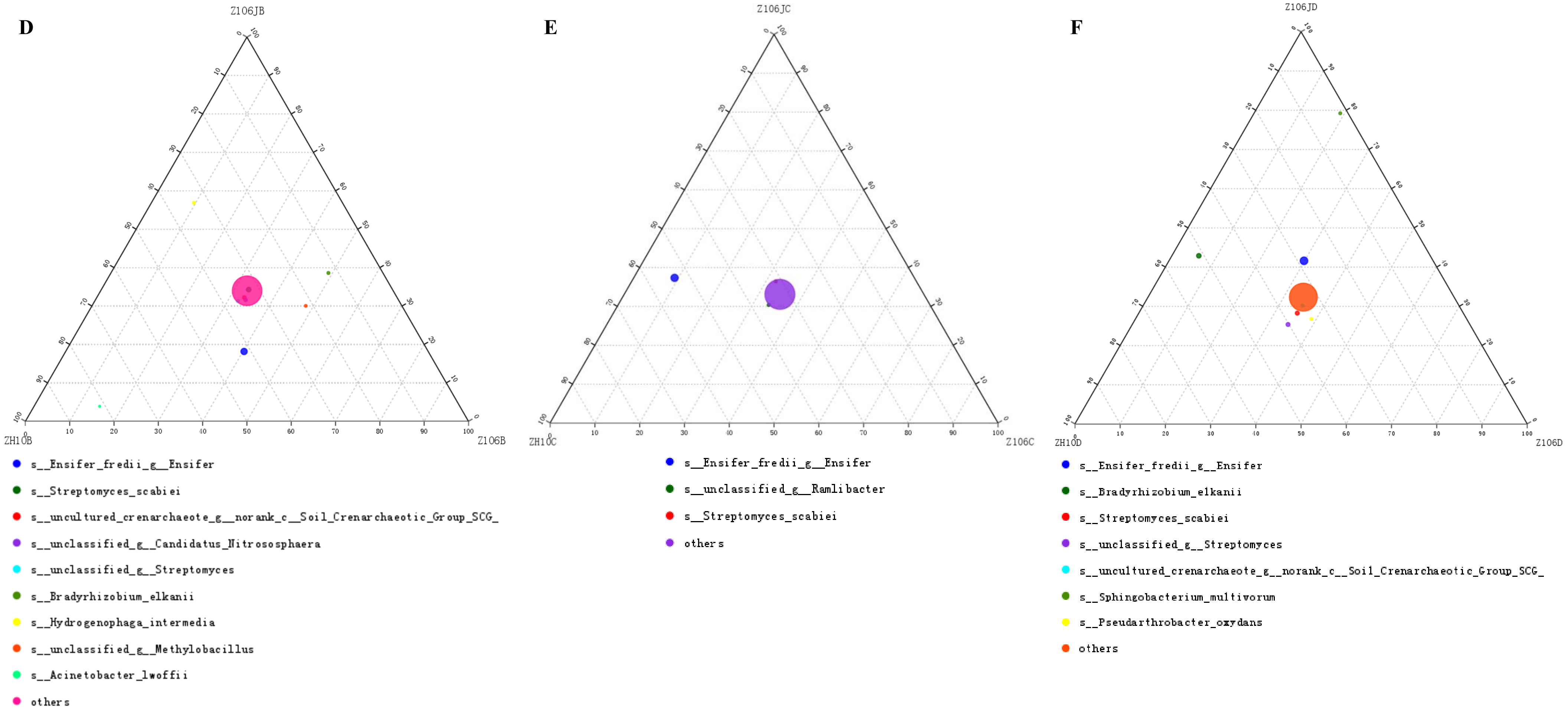

Fig. S7: Ternary plot of species based on different treatments in surrounding soil (A), rhizospheric soil (B), root (C) , seedling stage (D), flowering stage (E) and seed-filling stage (F).

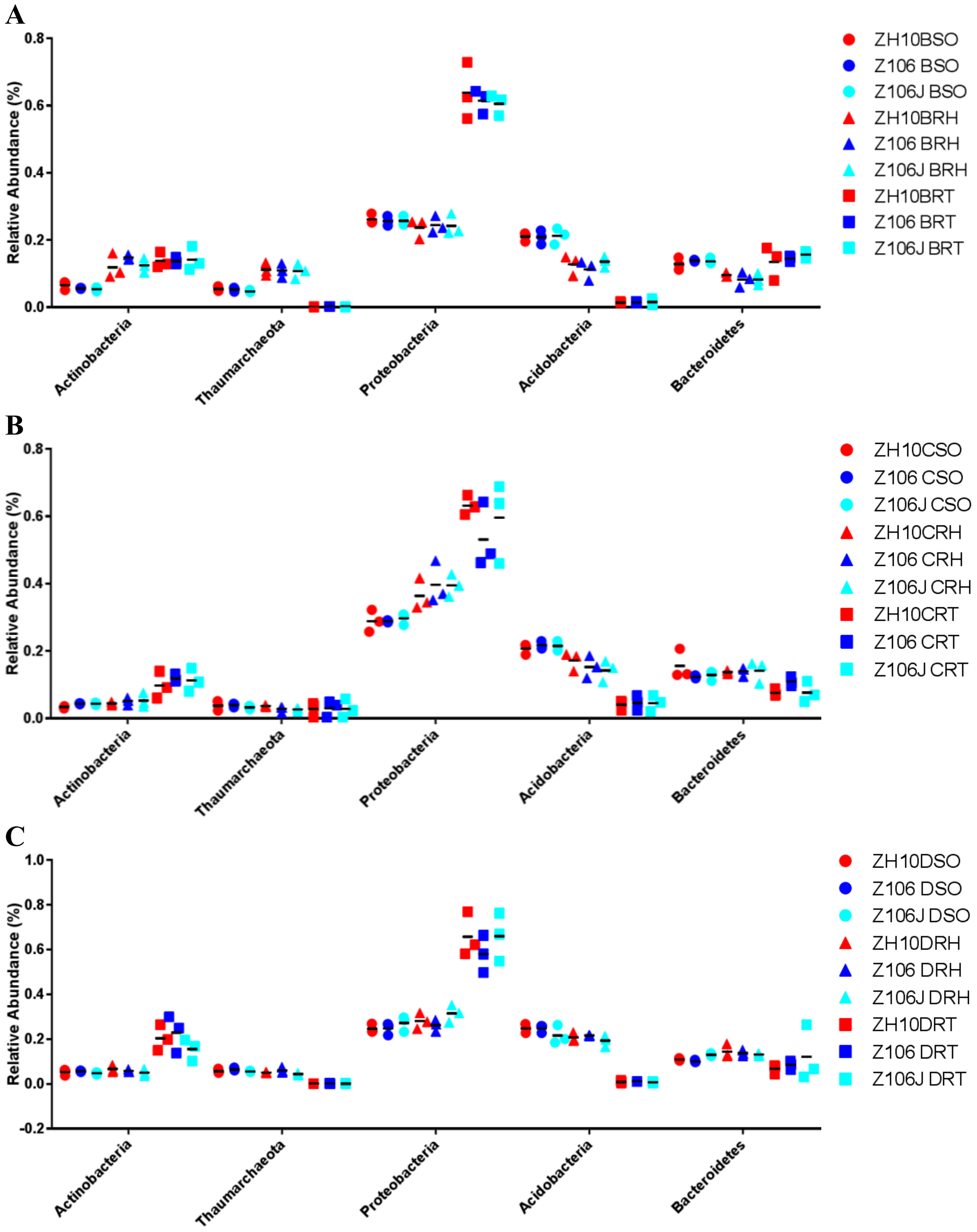
A
B
C
Relative Abundance (%)
Actinobacteria
Thaumarchaeota
Proteobacteria
Acidobacteria
Bacteroidetes
ZH10BSO
Z106 BSO
Z106J BSO
ZH10BRH
Z106 BRH
Z106J BRH
ZH10BRT
Z106 BRT
Z106J BRT
ZH10CSO
Z106 CSO
Z106J CSO
ZH10CRH
Z106 CRH
Z106J CRH
ZH10CRT
Z106 CRT
Z106J CRT
ZH10DSO
Z106 DSO
Z106J DSO
ZH10DRH
Z106 DRH
Z106J DRH
ZH10DRT
Z106 DRT
Z106J DRT

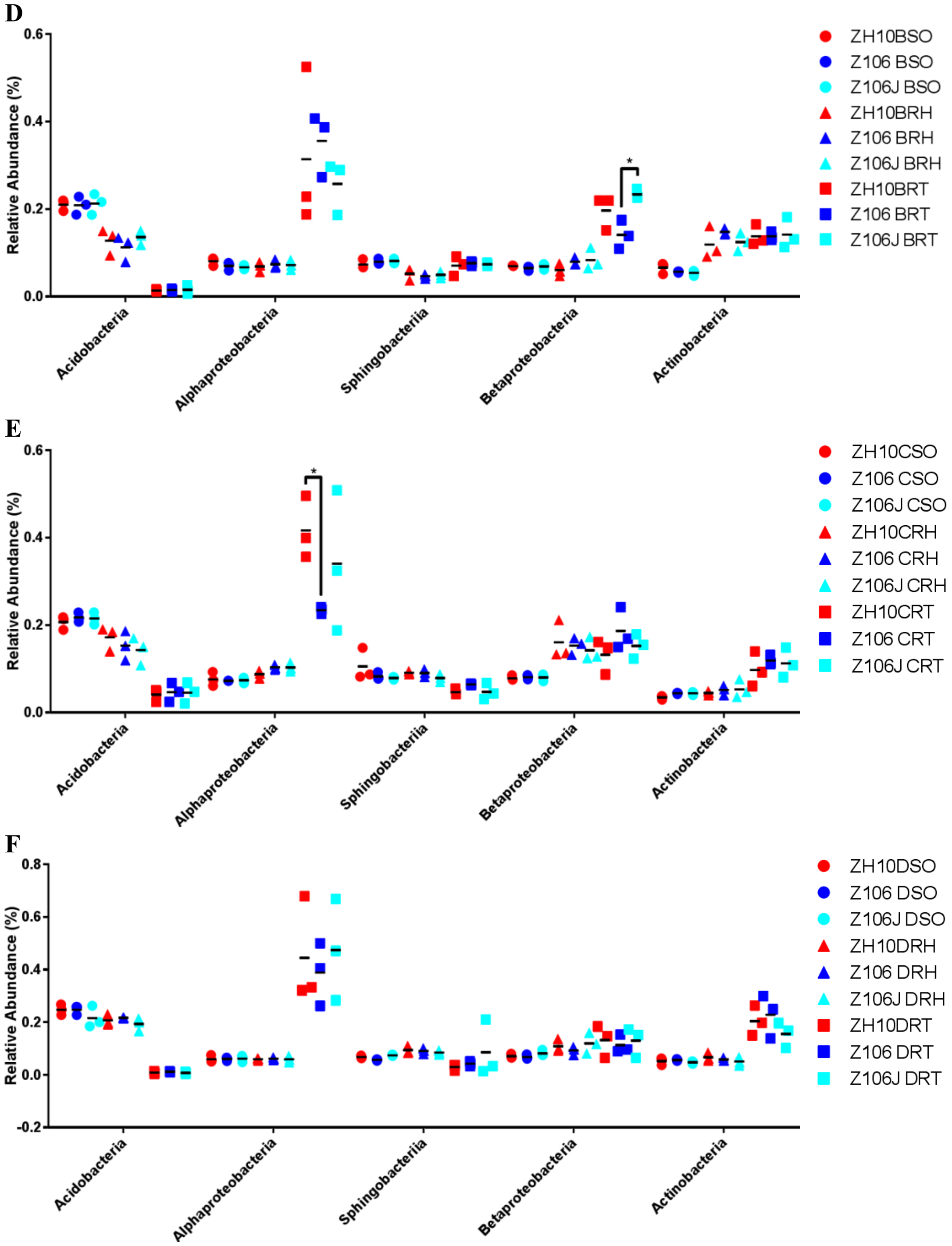
D
Relative Abundance (%)
0.6
0.4
0.2
0.0
*
Acidobacteria
Alphaproteobacteria
Sphingobacteriia
Betaproteobacteria
Actinobacteria
ZH10BSO
Z106 BSO
Z106J BSO
ZH10BRH
Z106 BRH
Z106J BRH
ZH10BRT
Z106 BRT
Z106J BRT
E
Relative Abundance (%)
0.6
0.4
0.2
0.0
*
Acidobacteria
Alphaproteobacteria
Sphingobacteriia
Betaproteobacteria
Actinobacteria
ZH10CSO
Z106 CSO
Z106J CSO
ZH10CRH
Z106 CRH
Z106J CRH
ZH10CRT
Z106 CRT
Z106J CRT
F
Relative Abundance (%)
0.8
0.6
0.4
0.2
0.0
-0.2
Acidobacteria
Alphaproteobacteria
Sphingobacteriia
Betaproteobacteria
Actinobacteria
ZH10DSO
Z106 DSO
Z106J DSO
ZH10DRH
Z106 DRH
Z106J DRH
ZH10DRT
Z106 DRT
Z106J DRT

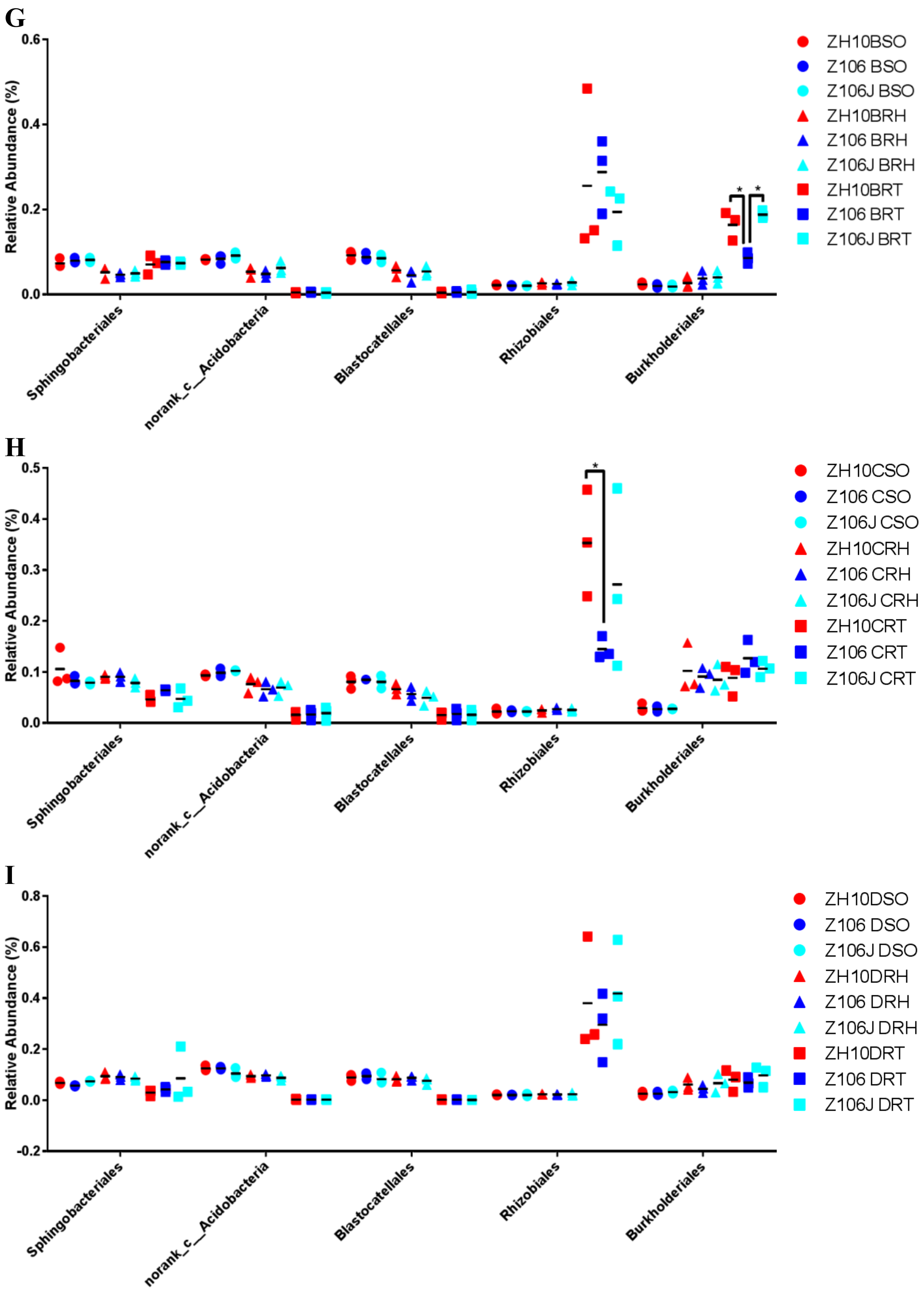
G
Relative Abundance (%)
0.6
0.4
0.2
0.0
Sphingobacteriales
norank_c__Acidobacteria
Blastocatellales
Rhizobiales
Burkholderiales
*
*
ZH10BSO
Z106 BSO
Z106J BSO
ZH10BRH
Z106 BRH
Z106J BRH
ZH10BRT
Z106 BRT
Z106J BRT
H
Relative Abundance (%)
0.5
0.4
0.3
0.2
0.1
0.0
*
Sphingobacteriales
norank_c__Acidobacteria
Blastocatellales
Rhizobiales
Burkholderiales
ZH10CSO
Z106 CSO
Z106J CSO
ZH10CRH
Z106 CRH
Z106J CRH
ZH10CRT
Z106 CRT
Z106J CRT
I
Relative Abundance (%)
0.8
0.6
0.4
0.2
0.0
-0.2
Sphingobacteriales
norank_c__Acidobacteria
Blastocatellales
Rhizobiales
Burkholderiales
ZH10DSO
Z106 DSO
Z106J DSO
ZH10DRH
Z106 DRH
Z106J DRH
ZH10DRT
Z106 DRT
Z106J DRT

J

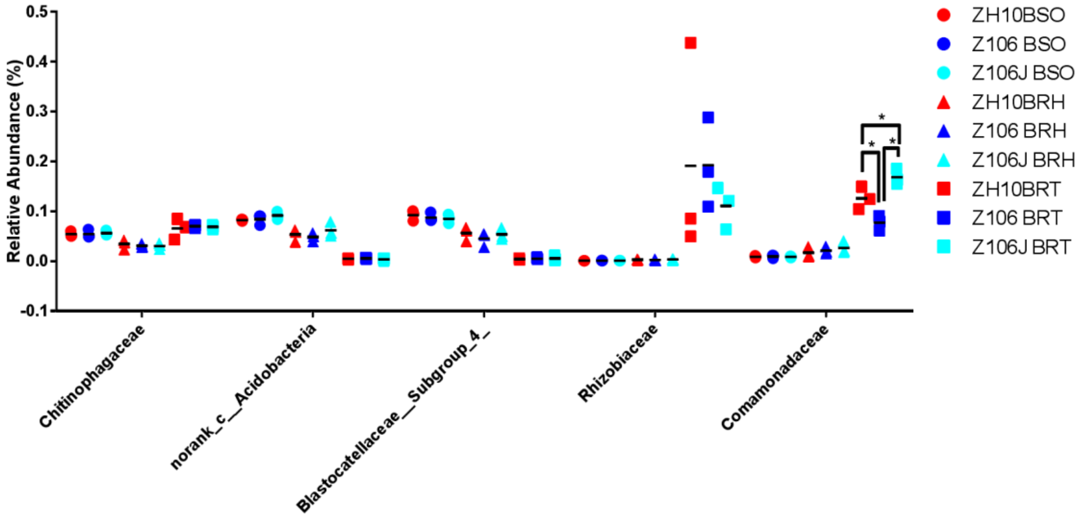
Relative Abundance (%)
0.5
0.4
0.3
0.2
0.1
0.0
-0.1
Chitinophagaceae
norank_c__Acidobacteria
Blastocatellaceae__Subgroup_4_
Rhizobiaceae
Comamonadaceae
*
ZH10BSO
Z106 BSO
Z106J BSO
ZH10BRH
Z106 BRH
Z106J BRH
ZH10BRT
Z106 BRT
Z106J BRT

K

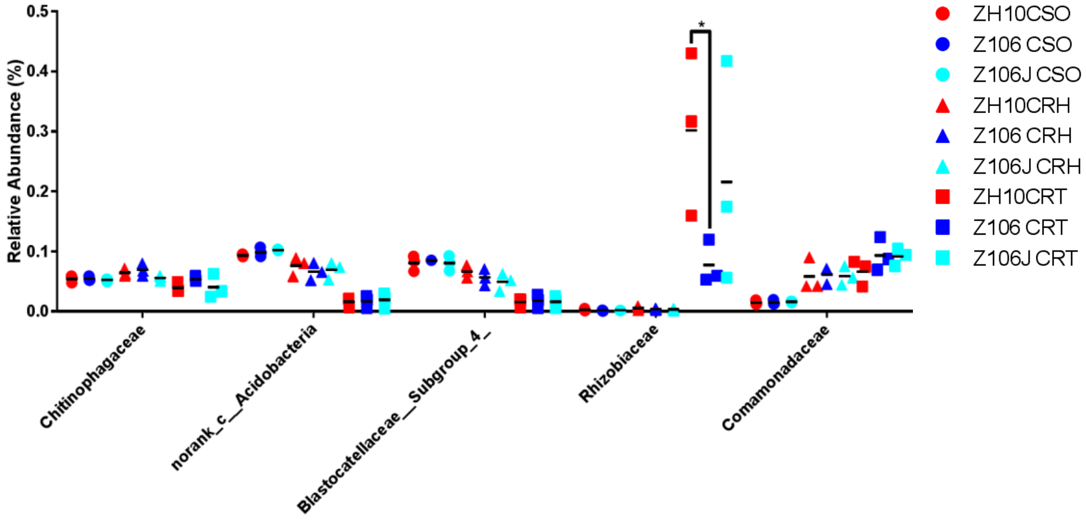
Relative Abundance (%)
0.5
0.4
0.3
0.2
0.1
0.0
Chitinophagaceae
norank_c__Acidobacteria
Blastocatellaceae__Subgroup_4_
Rhizobiaceae
Comamonadaceae
*
ZH10CSO
Z106 CSO
Z106J CSO
ZH10CRH
Z106 CRH
Z106J CRH
ZH10CRT
Z106 CRT
Z106J CRT

L

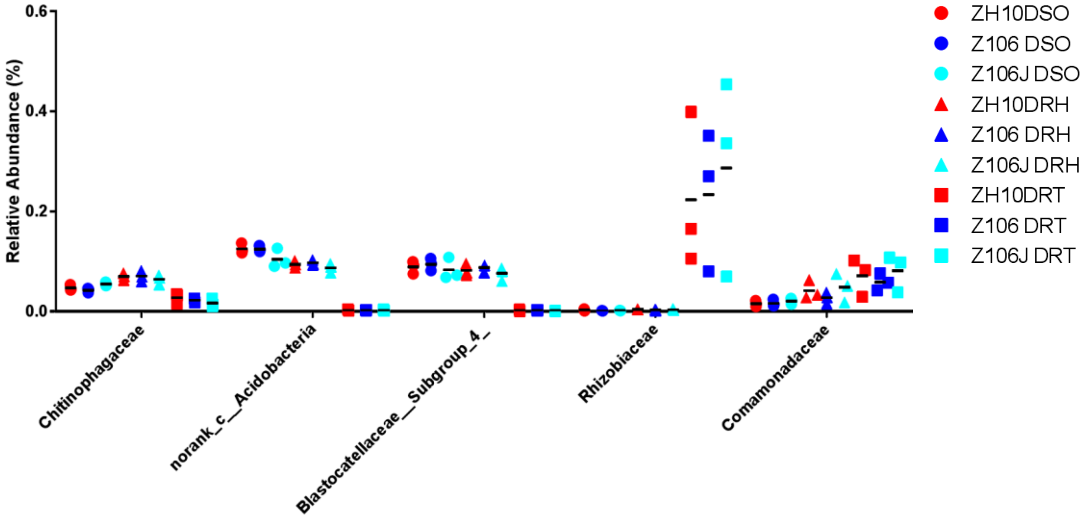
Relative Abundance (%)
0.6
0.4
0.2
0.0
Chitinophagaceae
norank_c__Acidobacteria
Blastocatellaceae__Subgroup_4_
Rhizobiaceae
Comamonadaceae
ZH10DSO
Z106 DSO
Z106J DSO
ZH10DRH
Z106 DRH
Z106J DRH
ZH10DRT
Z106 DRT
Z106J DRT

M

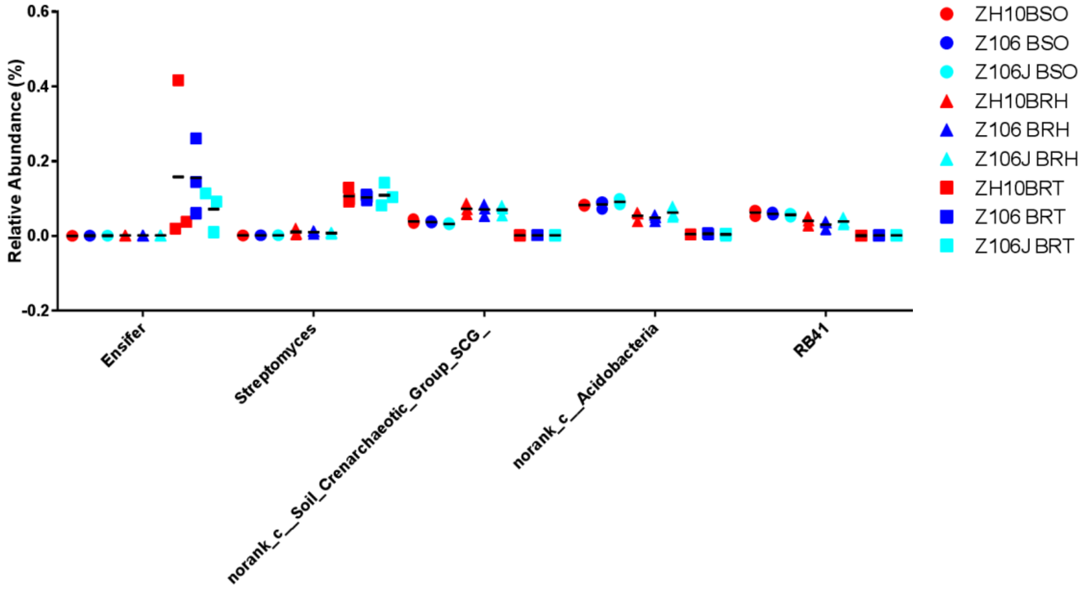
Relative Abundance (%)
0.6
0.4
0.2
0.0
-0.2
Ensifer
Streptomyces
norank_c__Soil_Crenarchaeotic_Group_SCG_
norank_c__Acidobacteria
RB41
ZH10BSO
Z106 BSO
Z106J BSO
ZH10BRH
Z106 BRH
Z106J BRH
ZH10BRT
Z106 BRT
Z106J BRT

N

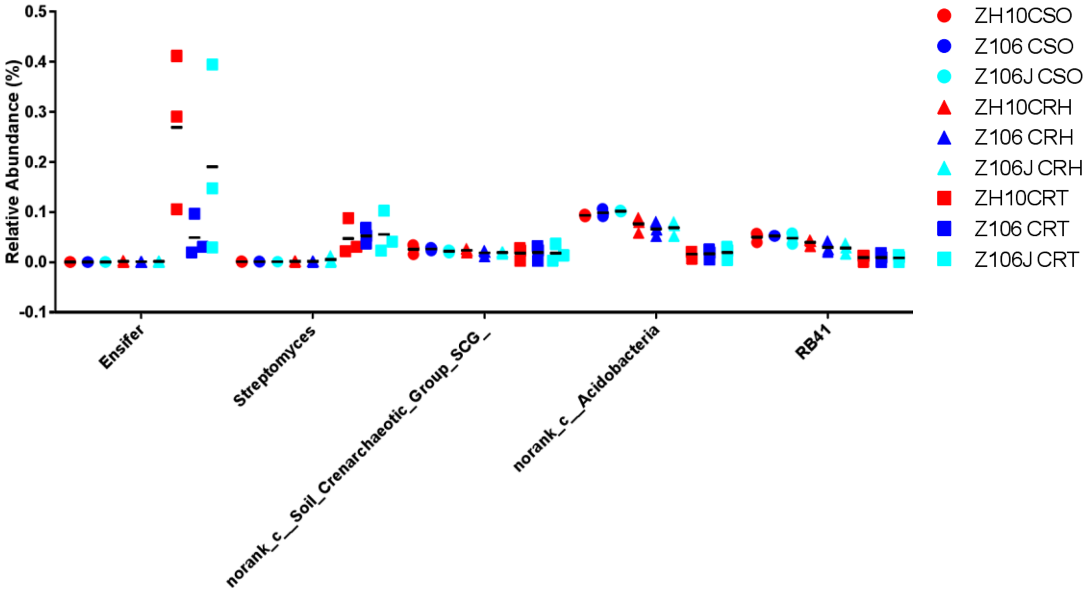
Relative Abundance (%)
0.5
0.4
0.3
0.2
0.1
0.0
-0.1
Ensifer
Streptomyces
norank_c__Soil_Crenarchaeotic_Group_SCG_
norank_c__Acidobacteria
RB41
ZH10CSO
Z106 CSO
Z106J CSO
ZH10CRH
Z106 CRH
Z106J CRH
ZH10CRT
Z106 CRT
Z106J CRT

O

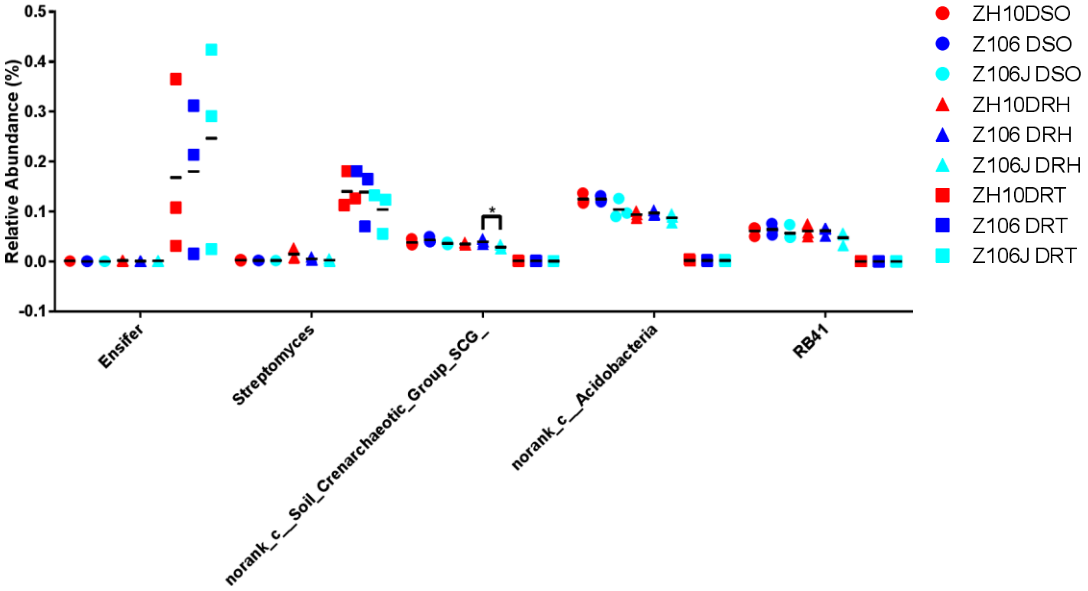
Relative Abundance (%)
0.5
0.4
0.3
0.2
0.1
0.0
-0.1
*
Ensifer
Streptomyces
norank_c__Soil_Crenarchaeotic_Group_SCG_
norank_c__Acidobacteria
RB41
ZH10DSO
Z106 DSO
Z106J DSO
ZH10DRH
Z106 DRH
Z106J DRH
ZH10DRT
Z106 DRT
Z106J DRT

P

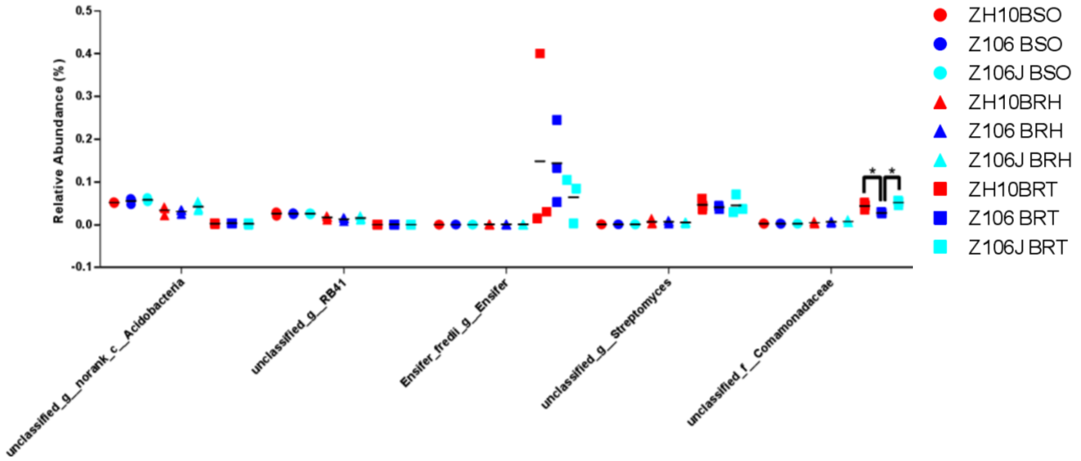

Q

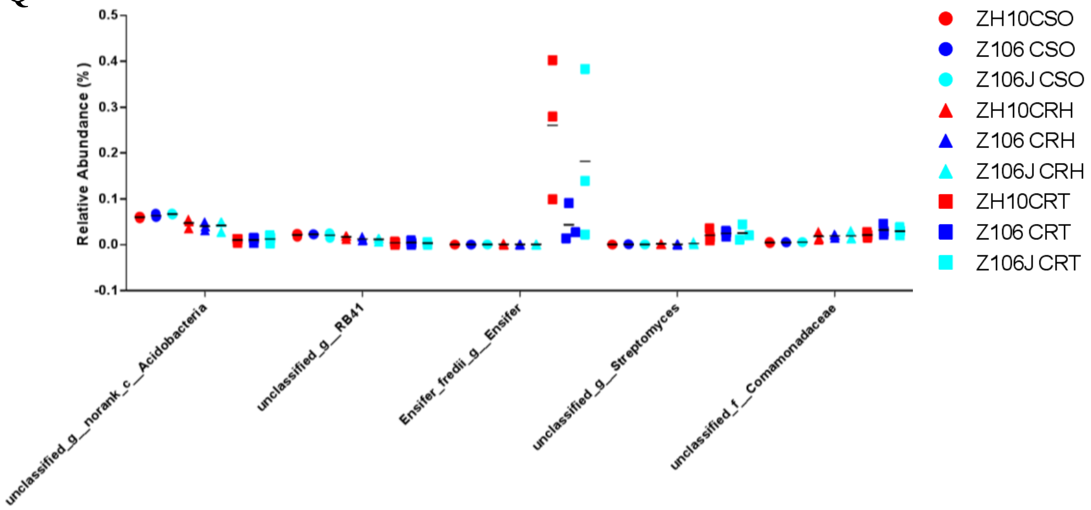

R

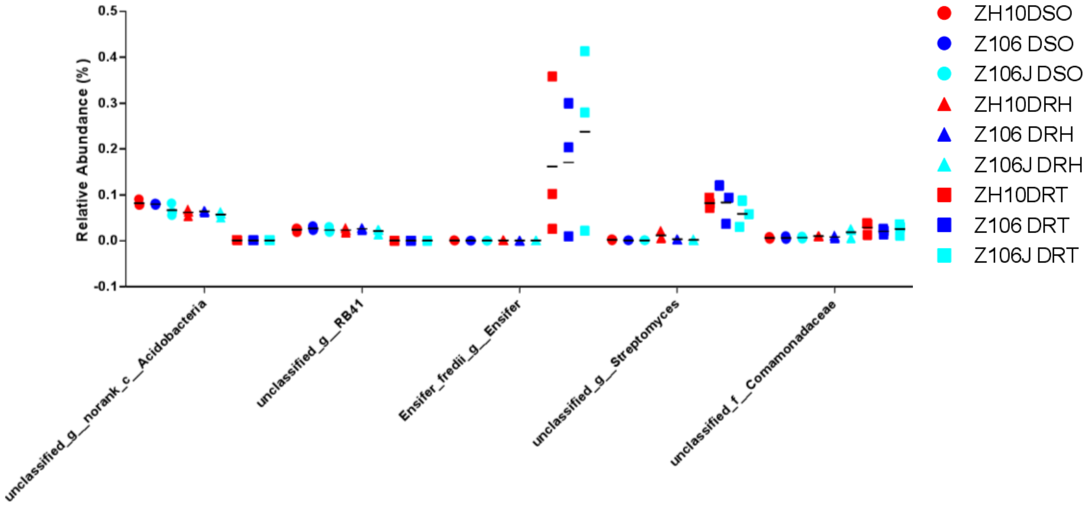

Fig. S8: The relative abundance scatter plot of the major five bacterial taxa among three treatments in different stages at Phylum (A, B, C), Class (D, E, F), Order (G, H, I), Family (J, K, L), Genus (M, N, O) and Species (P, Q, R) level.
